# A critical review of the neuroimaging literature on synesthesia

**DOI:** 10.3389/fnhum.2015.00103

**Published:** 2015-03-31

**Authors:** Jean-Michel Hupé, Michel Dojat

**Affiliations:** ^1^Centre de Recherche Cerveau et Cognition, Université de Toulouse and Centre National de la Recherche Scientifique, ToulouseFrance; ^2^Grenoble Institut des Neurosciences, Institut National de la Santé et de la Recherche Médicale U836 and Université Grenoble Alpes, GrenobleFrance

**Keywords:** synesthesia, fMRI, VBM, DTI, connectivity

## Abstract

Synesthesia refers to additional sensations experienced by some people for specific stimulations, such as the systematic arbitrary association of colors to letters for the most studied type. Here, we review all the studies (based mostly on functional and structural magnetic resonance imaging) that have searched for the neural correlates of this subjective experience, as well as structural differences related to synesthesia. Most differences claimed for synesthetes are unsupported, due mainly to low statistical power, statistical errors, and methodological limitations. Our critical review therefore casts some doubts on whether any neural correlate of the synesthetic experience has been established yet. Rather than being a neurological condition (i.e., a structural or functional brain anomaly), synesthesia could be reconsidered as a special kind of childhood memory, whose signature in the brain may be out of reach with present brain imaging techniques.

## INTRODUCTION

Synesthesia may be defined as the subjective phenomenon of additional experiences that sometimes, but not always, involves mixing sensory modalities: in some people, perceptual, emotional, or internally generated stimulation evokes sensory, representational, cognitive, or affective “synesthetic” experiences (Macpherson, 2007; Hupé et al., 2012b; Simner, 2012; Chun and Hupé, 2013a). Synesthetic associations are supplementary, arbitrary, idiosyncratic, and usually have an involuntary feel (they are not evoked at will and are not chosen, contrary to metaphors). They usually cannot be suppressed, so they are also often described as automatic (in a weak sense) or inevitable (Ward, 2013). Examples of types of synesthesia that fulfill this definition include not only canonical varieties like colored hearing, but also colored letters and numbers (graphemes), sequence-space (including number-lines: Galton, 1883), and personifications of numbers and letters (Flournoy, 1893). These three most frequent types are found in 1–10% of the population (Simner et al., 2006; Chun and Hupé, 2013a). We do not consider the experiences of mirror-touch (Blakemore et al., 2005) and ticker tape (where speech is experienced as subtitled in the mind’s eye) as types of synesthesia because in both cases associations are not arbitrary (Chun and Hupé, 2013a). Color is the most frequent additional experience (Day, 2005).

The present consensus is “that synesthesia is neither imagination nor is it metaphorical thinking, instead it has a neural basis” (Rothen et al., 2012). But what are the neural correlates of synesthesia? The subjective experience of synesthetic colors could activate brain regions normally responsible for the perception of real colors differently from when non-synesthetes just imagine or remember colors. Such neural activation could be due to extra neuronal connections, in particular from neighboring regions (cross-activation theory), or from a difference in neuronal transmission (disinhibited feedback theory). Several studies also suggested more distributed neuronal differences resulting in different brain network properties in synesthetes. In his thorough review on synesthesia, Ward states that “candidate neural mechanisms of synesthesia all have something in common insofar as they are believed to reflect differences in connectivity relative to the neurotypical brain. Moreover, these differences are typically assumed to lie at the cortical level, reflecting the complex nature of the inducer/concurrent” (Ward, 2013). This echoes a former statement by Blake et al. (2005) on grapheme-color synesthesia: “Virtually all neural models of synesthesia propose that it arises from an atypical pattern of connectivity between form processing and color processing centers of the brain,” with the difference in connectivity being structural or functional. In either case, the experience of synesthesia should induce neural activity in the regions normally involved in the experience of the concurrent (the induced association). That is, color centers should be activated when grapheme-color synesthetes read achromatic letters or words (the inducers), imagine them or listen to them.

Rouw et al. (2011) reviewed the studies that have searched for the neural correlates of synesthesia. They gathered the data interpretations proposed in neuroimaging studies on synesthesia, in search of an emergent consistent pattern. The logic behind such a meta-analysis is correctly based on the assumption that when effect sizes are small, they should not reach significance in every study because of sampling error, especially when tested groups are small, as is the case in the field of synesthesia. However, meta-analyses may be biased when the reported “significant” results are questionable. A quantitative meta-analysis of this magnetic resonance imaging (MRI) literature (e.g., Yarkoni et al., 2011) would require not only that similar protocols were used (that was not the case), but also to have access to effect sizes at each voxel, in order to include also the studies that *did not* find significant results. While several initiatives promote data sharing in open data sources (e.g., Poline et al., 2012; Keator et al., 2013; Sternberg et al., 2013), currently, in most studies information is given only about the coordinates of voxels that pass an arbitrary, often disputable, statistical threshold.

Here, we followed an approach quite different from usual meta-analyses of MRI data, adopting a systematic *skeptical* point of view in order to first deconstruct the results from the interpretation proposed by their authors, and then evaluate every dataset with similar and robust criteria. We therefore reexamined all studies that have searched for the neural correlates of synesthesia. In contrast to prevailing consensus within the synesthesia literature, and to the conclusion of Rouw et al. (2011), our analysis casts some doubt on whether any cortical marker of synesthesia has yet been discovered, and we suggest, based on the present data, that synesthesia may be not due to some brain alteration. We could therefore reconsider synesthetic associations as simply memories of a special kind. The neural correlates of synesthesia may be difficult to identify as long as detecting the signature of memory contents in the brain remains beyond the reach of current brain imaging techniques.

Part 1 is devoted to functional studies, where we review in particular the empirical evidence in support of color centers being activated in color synesthesia. Part 2 reviews the available evidence in favor of structural brain differences between synesthetes and controls. We reviewed studies on developmental synesthesia, not acquired synesthesia. We tried to be as exhaustive as possible, including the few EEG studies on the subject. The paper is organized around color-induced synesthesia, because of its large dominance in the literature. Additionally, we reviewed the few studies on other types on synesthesia. A critical summary of each cited study appears in the Appendix. A companion paper contains a methodological explanation of the statistical issues that guided our reading and interpretation of each paper (Hupé, 2015); readers not familiar with the subtleties of statistical inference or MRI methods are invited to consult it.

## PART 1. FUNCTIONAL NEURAL CORRELATES OF SYNESTHESIA

**FIGURE 1:**
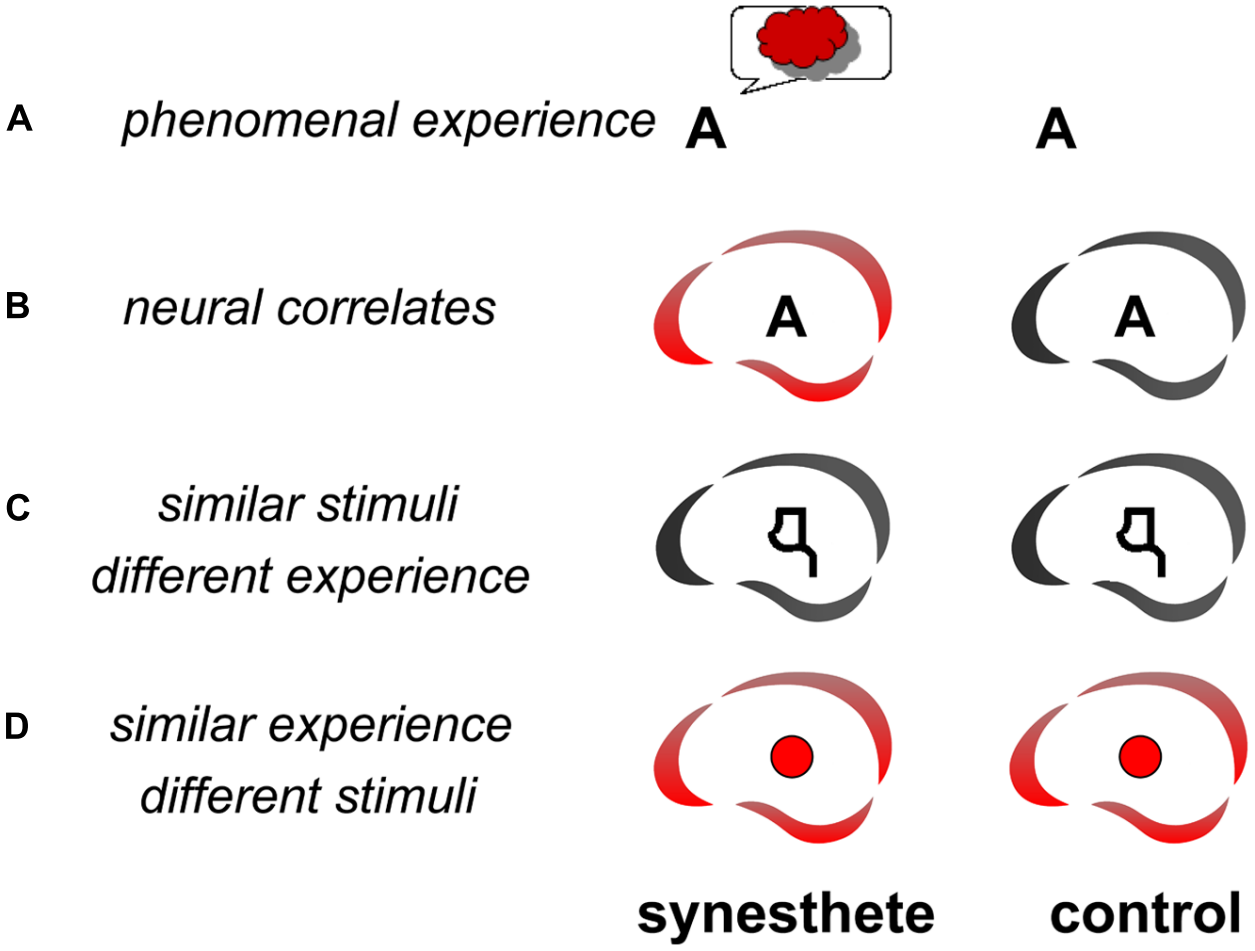
**Methodology for searching the functional neural correlates of synesthesia.** The letter A may trigger the experience of red for a synesthete but not a control subject **(A)**. In order to isolate the neural correlates (represented as the brain icon of the CerCo lab logo) of this experience, one may compare the brain activations of synesthetes and controls to this stimulus **(B)**. To ensure that any observed difference is due to the synesthetic experience, one may compare the same subjects for similar activations that do not trigger any synesthetic experience, like pseudo-letters or false fonts **(C)**. In such a control experiment, the stimuli are therefore “similar” to those triggering the synesthetic experience, but the phenomenal experience is “different” than the synesthetic experience. One may also compare the activation by synesthetic and real colors **(D)**.

Several studies have looked for the neural correlates of the synesthetic experience (**Figure 1A**). The question is: “what brain areas are uniquely activated when an individual has a synesthetic experience?” (Blake et al., 2005). Finding the answer is not straightforward, because the neural correlates of the synesthetic experience need to be dissociated from the neural correlates of the inducer. There are three complementary ways to resolve this question: (1) Compare the brain activity of synesthetes and non-synesthetes to the same stimuli (**Figure 1B**); (2) Compare the brain activity in synesthetes for similar stimuli that induce or do not induce synesthesia (**Figure 1C**); (3) Compare the brain activity of synesthetes for synesthetic versus non-synesthetic experiences that are similar (e.g., synesthetic vs. real colors: **Figure 1D**). At least two of these comparisons (three possible combinations) should be convergent in order to identify a candidate area as a neural substrate of the synesthetic experience. For example [comparisons (1) and (2), Section 1.1. below], if an area is found to be differently activated in synesthetes and controls (**Figure 1B**), it would remain to be shown that the differential activity is directly related to the synesthetic experience (**Figure 1C**) and not to secondary characteristics, such as differences in attention, emotion, or imagery. These characteristics may need to be included to fully describe the synesthetic experience but they are not specific to the experience of synesthesia.

Most studies used functional magnetic resonance imaging (fMRI) and tested grapheme-color synesthesia, sometimes considered as colored hearing when the stimuli (words) are presented in the auditory modality. Our main question is whether any functional correlate of the synesthetic experience has been identified yet. Our secondary question is whether these functional correlates are the same as those involved in a comparable non-synesthetic experience (e.g., are color centers activated when synesthetes experience colors for achromatic stimuli?).

### 1.1. SYNESTHETES vs. CONTROLS FOR STIMULI INDUCING SYNESTHESIA OR NOT (13 STUDIES)

We identified one PET and eight fMRI studies that compared groups of grapheme-color synesthetes and controls (**Table 1**, first section). The synesthetic experience of color was elicited either by letters presented visually (Hubbard et al., 2005a; Rouw and Scholte, 2007, 2010; van Leeuwen et al., 2010; Sinke et al., 2012; O’Hanlon et al., 2013; Tomson et al., 2013) or by words presented in the auditory modality (Paulesu et al., 1995; Gaschler-Markefski et al., 2011). We shall also report in this section the results of four more studies on different types of synesthesia (**Table 1**, second section: Bor et al., 2007; Tang et al., 2008; Jones et al., 2011; Neufeld et al., 2012a). We did not include here three other fMRI studies that tested groups of synesthetes and controls but did not report the statistical comparison between both groups (**Table 1**, first section: Nunn et al., 2002; Gray et al., 2006; Rich et al., 2006). These studies are considered in Section 1.2.

**Table 1:** MNI XYZ coordinates (mm) of left parietal regions.

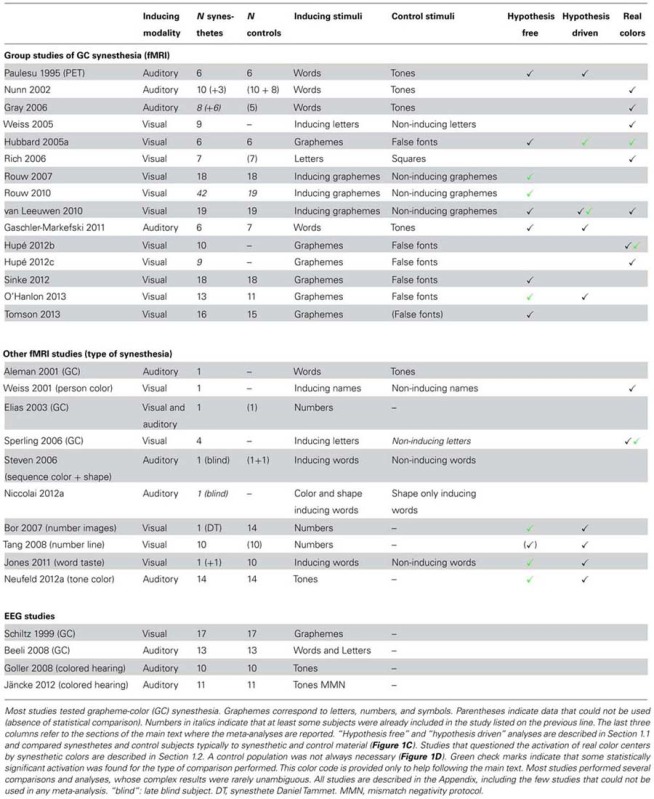

#### Hypothesis free analysis of group studies for colored synesthesia (nine studies)

First we evaluate the nine group studies of colored synesthesia without making any hypothesis about where in the brain differences should be observed (**Table 1**, column “Hypothesis free”). We consider differences as significant only when statistical procedures to control the risk of false positives among multiple comparisons over the whole brain were applied, or when applying two-tailed tests for region of interest (ROI) analyses. Differences between synesthetes and controls when comparing the responses to synesthetic and control stimuli survived these strict criteria in only three studies (green check marks in **Table 1**). Group sizes (synesthetes/controls) in the studies that did not find significant differences were 6/6 (Paulesu et al., 1995), 6/6 (Hubbard et al., 2005a), 19/19 (van Leeuwen et al., 2010), 6/7 (Gaschler-Markefski et al., 2011), 18/18 (Sinke et al., 2012), and 16/15 (Tomson et al., 2013). Results in the three studies that found significant differences were not consistent. Different statistical contrasts were used and no single anatomical region was common to any two studies, despite the fact that the induced synesthetic experience always involved color. A synthesis of the results reported in these three studies is as follows.

Rouw and Scholte (2007, 2010) compared synesthetes and controls for the contrast between graphemes (digits, letters, or symbols) that elicited strong or weak synesthetic colors and graphemes that did not. This contrast may correspond to the activation by synesthetic colors only in synesthetes. They measured greater activation for synesthetes in four regions in their 2007 study and in three regions in their 2010 study (no region showed greater activation in controls than in synesthetes in either study). These regions did not seem to overlap even though the 2010 study (42 synesthetes against 19 controls) included all subjects of their 2007 study (18 synesthetes against 18 controls). The only significant difference between synesthetes and controls observed in the visual cortex in 2007 (in the fusiform gyrus, therefore close to color regions), was not found in the 2010 study. Rouw and Scholte performed only group comparisons. They did not report whether the blood-oxygen-level-dependent (BOLD) signal for synesthetes was *significantly* stronger for synesthetic than non-synesthetic graphemes in any of these regions, or in any region at all. The general difference between synesthetes and controls (corresponding to an interaction effect) could be due in part to weaker responses to non-eliciting graphemes in synesthetes than in controls, a result not easily interpretable (the experimental design supposed that non-synesthetic graphemes were perceived similarly in synesthetes and non-synesthetes and induced similar brain activity).

Using a similar protocol (graphemes and false-fonts), O’Hanlon et al. (2013) tested all possible interaction effects between group (13 synesthetes vs. 11 controls) and stimuli. They identified 14 brain regions with significant interaction effects, none of which had a stronger response to graphemes in synesthetes, but two with a stronger activation in controls (none in the visual cortex; they also observed group differences for false-fonts).

#### Hypothesis free analysis (four additional studies)

Two studies compared a control group with a single synesthete, one [Daniel Tammet (DT)] for complex mental images evoked by numbers (Bor et al., 2007) and the other for tastes experienced with words (Jones et al., 2011). Bor et al. (2007) detected significant differences in BOLD signal between DT and 14 controls only in the frontal cortex (tested during a digit span task, with no control condition comparable to other studies). When comparing the BOLD signal for “tasty” and “tasteless” words, Jones et al. (2011) found a significant difference between a synesthete and 10 controls only in the precuneus. However, this region was not significantly more activated by tasty than tasteless words in the whole brain analysis when performed in the synesthete alone. Note that classical statistical models may not be appropriate when comparing one subject to a group: the results critically depend on the construction of the group and may require very large control groups (e.g., Nocchi et al., 2008).

Tang et al. (2008) and Neufeld et al. (2012a) compared non-synesthetes to, respectively, a group of 10 synesthetes with number form and a group of 14 tone-color synesthetes, but they did not include any control stimulus. Tang et al. (2008) focused on interaction effects for different tasks that all involved synesthetic stimuli. When contrasting numbers that evoked number lines to baseline in the group of synesthetes alone (they did not report the comparison with controls), these stimuli did not evoke any significant response in the posterior intraparietal sulcus (IPS), the region previously argued to be involved in spatial processing (Hubbard et al., 2005b). Neufeld et al. (2012a) measured a stronger activation to tones for synesthetes compared to controls in the left inferior parietal cortex (they performed conservative voxelwise statistics with additional cluster extent threshold), but this activation lacked specificity (tones were contrasted against baseline). Rouw and Scholte (2010) had also reported a significant cluster in the parietal cortex. It was, however, 10 times larger and the MNI coordinates of the cluster centers in the two studies were several centimeters apart (**Table 2**); the cluster extent statistics used by Rouw and Scholte do not indicate, anyway, where the difference is within the cluster (see Hupé, 2015: third section “Pitfalls of MRI statistics”).

**Table 2:** MNI XYZ coordinates (mm) of left parietal regions.

	fMRI BOLD signal *Synesthetes > Controls*	VBM GM *Synesthetes > Controls*	Connectivity *Seeds used*
Weiss 2005	[-36, –54, 41]^(1)^ [-24, -70, 52]^(2)^		
Weiss 2009		[-24, –64, 47]^(3)^	
Rouw 2010	[-30, -72, 28]^(4)^ [-3, -75, 30]^(5)^	[-11, -58, 61]^(6)^	
Neufeld 2012a	[-46, -54, 58]^(7)^		
Neufeld 2012b			[-46, -54, 58]
Sinke 2012	[-36, -62, 48]^(8)^		[-36, –62, 48]
Jäncke 2011			[-20, -65, 50]
van Leeuwen 2010	[-24, -58, 46]^(9)^		
van Leeuwen 2011			[-24, -58, 46]

*^(1)^*“*Anterior intraparietal cortex” (fixed effect analysis). Published coordinates in Talairach space converted to MNI space.*

*^(2)^“Caudal intraparietal cortex” (fixed effect analysis).*

*^(3)^pSVC < 0.05. Small volume centered on the coordinates of Weiss 2005.*

*^(4)^“Intraparietal sulcus.” Cluster extent = 3280 mm^*3*^.*

*^(5)^“Parieto-occipital sulcus.” Cluster extent = 4656 mm^*3*^.*

*^(6)^“Superior parietal cortex.” Uncorrected. Cluster extent = 2944 mm^*3*^.*

*^(7)^“Inferior parietal cortex.” The contrast was *tones* > *scanner noise*. Cluster extent = 328 mm^3^ (41 voxels).*

*^(8)^“Inferior parietal lobule.” The contrast was not specific: *Synesthetes*≠ *Controls* for either letters or false fonts.*

*^(9)^“Superior parietal lobule.” The contrast was not specific: *Synesthetes* > *Controls* for black inducing graphemes > colored non-inducing graphemes.*

#### Conclusion of the hypothesis free analysis

Among 12 studies (out of 13: Tang et al., 2008, did not report this comparison) that tested whether differences exist between synesthetes and controls for synesthetic stimuli, only six observed significant differences, most of them in the frontal and parietal cortex, with no consistency across results. The three *group* studies (out of nine) that reported significant interactions between participant type (synesthete vs. non-synesthete) and stimulus type (synesthesia inducing vs. control) computed cluster extent statistics apparently using state-of-the-art methods. However, they did not seem to apply optimal parameters. Rouw and Scholte (2007, 2010) used a low cluster-forming threshold (*z* = 2.3) and O’Hanlon et al. (2013) smoothed their data only slightly [7 mm full width at half maximum (FWHM)], tested a small size population (13 synesthetes and 11 controls), and did not specify how they computed Monte Carlo simulations in order to set the significance threshold. These choices may lead to a high rate of false positives^1^ (Silver et al., 2011) and could explain the inconsistencies in brain regions and direction of effect. O’Hanlon et al. (2013) also reported stronger responses to letters in non-synesthetes (possibly corresponding to deactivation by synesthetic colors in synesthetes) that were not systematically tested in the other studies.

Since stimuli were designed to trigger a concurrent sensory experience specifically in synesthetes, the most surprising result across these 13 studies was the absence of significant difference (with the possible exception of Rouw and Scholte, 2007) between synesthetes and controls in the visual cortex (or gustatory cortex or posterior IPS), especially in the regions thought to be involved in the experience of the concurrent, which was color in 10 studies (retinotopic area V4 and anterior regions of the fusiform gyrus), at least when exploring the data with no *a priori* hypothesis.

#### Hypothesis driven analysis

The absence of significant difference is of course not the proof of the absence of difference, especially when considering that these studies had low power. But nine of these studies also documented the responses measured in the regions of the concurrent experience (mostly color regions; for the four other studies, only whole brain analysis was performed: Rouw and Scholte, 2007, 2010; Sinke et al., 2012; Tomson et al., 2013; see **Table 1**, column “Hypothesis driven”). As explained in (Hupé, 2015: second Section “Common mistakes with statistical inference,” paragraph 4), if adjusting the statistical threshold by making a specific hypothesis (that some activation should be observed within a given region, e.g., a color region), the observed results cannot validate the hypothesis made to obtain them (that would be circular reasoning). The only inference in such a study could concern where in this region activation is the most likely (if one accepts the hypothesis). But exploring data at a lenient threshold in several studies may reveal consistent effects whose size is too small to reach significance in each study.

Two studies obtained results compatible with the involvement of color regions in synesthesia. Hubbard et al. (2005a) measured a stronger BOLD response in retinotopically defined V4 for six synesthetes, compared to six controls, when contrasting characters to false fonts. This difference was just significant (*p* < 0.05) with a one-tailed test. van Leeuwen et al. (2010) measured the difference in BOLD response to inducing and non-inducing graphemes within a color ROI in the anterior fusiform gyrus that was previously obtained by contrasting colored versus black letters [the peak activation was “within 5 mm of the reported anatomical location of anterior visual area V4α”; they corrected the statistical risk for the number of comparisons in the ROI, using a “Small Volume Correction” (SVC) procedure]. The response to synesthetic stimuli was stronger in 19 synesthetes compared to 19 controls (cluster of 21 voxels in the right fusiform gyrus, pSVC = 0.052).

Six studies found no significant differences. Using PET, Paulesu et al. (1995) observed no statistical difference (uncorrected *p* > 0.05) between 6 synesthetes and 6 controls when contrasting words to tones at the Talairach coordinates of V4 as defined by Zeki et al. (1991). van Leeuwen et al. (2010) also measured, in their second experiment (adaptation protocol), the difference in BOLD response to inducing and non-inducing graphemes within their color ROI. They used a different sequence of stimuli but the same subjects. They no longer observed any significant difference between participants. In visual cortex ROIs (occipital lobe and fusiform gyrus), Gaschler-Markefski et al. (2011) did not observe stronger BOLD response in six synesthetes compared to seven controls for color-inducing words, compared to tones (uncorrected *p* > 0.05, one-tailed test). The ROI analysis by Neufeld et al. (2012a) using the V4 coordinates of McKeefry and Zeki (1997) did not reveal any difference (the statistical threshold was not reported). Bor et al. (2007) similarly reported that all *p*-values were above 0.1 for their ROI analyses, including V4 (coordinates from Nunn et al., 2002). With their word-taste synesthete, Jones et al. (2011) compared the response to “tasty” and “tasteless” words in the left anterior insula (“taste” ROI) and found no significant difference between the synesthete and the 10 controls (pSVC > 0.05). Tang et al. (2008) did not observe any significant activation at a lenient threshold (voxelwise uncorrected *p* < 0.001) in the posterior IPS, when contrasting numbers to baseline in their group of 10 number-line synesthetes.

Finally, one study reported a result in the opposite direction to the experimental hypothesis that synesthetes should show greater activation in sensory areas related to their concurrent experience. O’Hanlon et al. (2013) compared the BOLD responses to graphemes and false-fonts in 13 synesthetes and 11 controls, in ROIs based on structural gray matter (GM) differences, four of them in visual regions (left lateral occipital cortex/fusiform gyrus, occipital cortex/fusiform gyrus bilaterally, and right lingual gyrus). The interaction between stimulus type and participant group was significant in the left occipital fusiform/lingual gyrus (uncorrected *p* = 0.034), with a weaker response to letters than false fonts, which was also weaker in synesthetes than controls (*p* = 0.141).

#### Conclusion of the hypothesis driven analysis

Using *a priori* hypotheses that synesthetic experience should correlate with sensory activation in the brain regions that are associated with corresponding non-synesthetic perceptual sensation, only 2/9 studies (Hubbard et al., 2005a; van Leeuwen et al., 2010) suggested the involvement of color regions in synesthesia. We should also recall that Rouw and Scholte (2007) measured greater activation in the right fusiform gyrus for synesthetic graphemes in 18 synesthetes compared to 18 controls (whole brain analysis). Anatomical location was, however, not consistent across these three studies. The center of gravity of the significant cluster reported by Rouw and Scholte (2007) was 1.5 cm lateral to typical V4 coordinates (probed by Hubbard et al., 2005a) and 1.8 cm away from the peak coordinates by van Leeuwen et al. (2010). Rouw and Scholte (2007) and van Leeuwen et al. (2010) did not report whether the contrast between synesthetic and non-synesthetic graphemes revealed any significant activation when considering only the group of synesthetes. In the study by Hubbard et al. (2005a), responses were significantly stronger for graphemes than false fonts all over the retinotopic areas of the visual cortex and for both synesthetes and controls, while O’Hanlon et al. (2013) observed no brain region showing stronger responses to graphemes; if anything, responses to false fonts were stronger. Under the Null hypothesis, “significant” effects are expected from time to time when strict control over the rate of false positives is not applied. This is quite precisely what we observe, with no systematic pattern of significant effect regarding the direction of the effect (when tested), as well as no anatomical overlap.

### 1.2. DOES THE EXPERIENCE OF SYNESTHETIC COLORS INVOLVE COLOR CENTERS? REAL vs. SYNESTHETIC COLORS (10 STUDIES, INCLUDING TWO STUDIES FROM THE PREVIOUS SECTION)

The studies presented below compared the brain activity in synesthetes for colored stimuli vs. stimuli inducing synesthetic colors (see **Table 1**, column “Real colors”).

#### Overlap of significant activation (six studies)

These studies tested whether there was any activation overlap when contrasting colored against achromatic stimuli and when contrasting synesthetic and control stimuli. Five studies did not find any overlap; one study had an ambiguous result.

Three studies performed whole-brain analyses. In a single-case study, Weiss et al. (2001) did not observe any overlap for colored and synesthetic visual stimuli. Nunn et al. (2002) observed stronger activation for heard words than tones in the left infero-temporal cortex of 10 synesthetes. They also found activation by colored Mondrians at about the same coordinates but in another group of subjects (non-synesthetes). However, they did not observe any significant activation by colored Mondrians in this area in a group of six synesthetes. Gray et al. (2006) measured a bilateral activation by colored Mondrians in another group of eight synesthetes; again, there was no overlap with the activation by heard words.

Three more studies constrained their analysis to regions of the visual cortex involved in the processing of real colors and, therefore, had more power. Weiss et al. (2005) defined an ROI in the fusiform gyrus, of 10 mm radius, around the peak activation measured for real colors in nine synesthetes. The most significant voxel, from a contrast between synesthesia inducing vs. non-inducing letters, reached only pSVC = 0.073 and was 9 mm away from the peak activation by real colors. Rich et al. (2006) observed a similar result within a larger ROI defined in seven synesthetes, with left activation by synesthetic letters 2 cm away from V4 coordinates (pSVC = 0.008). Note that the control stimulus was not very specific (squares). Sperling et al. (2006) compared the activation by real colors and letters within retinotopic V4 in four synesthetes (single-subject studies). In two of them, they identified a few V4 voxels that responded significantly (compared to baseline) to colored Mondrians, as well as to letters inducing synesthetic colors. Response to letters inducing gray/transparent (not colored) experiences was weaker than to inducing letters, possibly significantly so (ambiguous report of the results).

#### ROI analysis (two studies)

Only two studies so far measured the average BOLD responses to inducing letters and false fonts in retinotopic V4 (Hubbard et al., 2005a; Hupé et al., 2012b), as well as in retinotopic areas V1, V2, V3, and V3a. Hubbard and colleagues observed in six synesthetes a significantly stronger response to letters, as well as to colored Mondrians, in all retinotopic areas (except V3a). Hupé and colleagues obtained similar results in 10 synesthetes; they also measured the average responses to letters and false fonts in individual ROIs of maximal response to colored Mondrians and found no significant difference.

#### Adaptation protocol (two studies)

The main issue for the interpretation of all previous studies is the necessary comparison to a control stimulus that does not elicit any synesthetic color. However, such a stimulus also differs by other properties. Adaptation protocols are not facing this problem. Adaptation (repetition/suppression) to stimuli with synesthetic and real colors can happen only if both experiences share neural correlates. van Leeuwen et al. (2010) measured the BOLD responses to letters followed by a colored square whose color was the same as or different than the synesthetic color of the letter. No adaptation was observed for congruent stimuli within six color ROI (where they had observed adaptation or strong responses to colored stimuli). Hupé et al. (2012c) obtained a similar Null result in the regions of maximal response to colored stimuli as well as in retinotopic areas.

#### Conclusion: synesthetic colors in color centers

Among 10 studies, only the three (if including Sperling et al., 2006) that performed retinotopic mapping observed stronger responses to both colored stimuli (than to gray stimuli) and to letters inducing synesthetic colors (than to false fonts). These results are compatible (but see the following discussion section) with the coding of both real and synesthetic colors in retinotopic areas, in particular V4 (but not in color centers), even though real and synesthetic colors do not seem to depend on the same neurons (no adaptation across colored and synesthetic stimuli). The other studies did not observe any overlap of activation for synesthetic and real colors.

### 1.3. COMPARISON OF SYNESTHETES AND NON-SYNESTHETES TO THE SAME STIMULI (INDUCING COLORS ONLY IN SYNESTHETES). ARE DIFFERENCES OBSERVED IN COLOR REGIONS? (FOUR EEG STUDIES)

Four EEG group studies (Schiltz et al., 1999; Beeli et al., 2008; Goller et al., 2008; Jäncke et al., 2012) compared the signals in synesthetes and controls for stimuli inducing synesthetic colors (**Table 1**, third section). Three of them used auditory stimuli and tested if stronger activation was observed over the visual cortex of synesthetes. Results were variable and not fully consistent even within each study. Jäncke et al. (2012) designed a particularly clever mismatch negativity task (MMN) to dissociate the magnitude of tone deviance from the magnitude of deviance evoked by synesthetic colors. Unfortunately, they did not manage to find synesthetes having associations tuned enough to this subtle design (please refer to the Appendix, Functional studies: EEG, MEG, for details and explanations).

## Discussion (Part 1)

Among 25 studies, we did not find any clear correlate of synesthetic colors. A few significant differences (in six studies) between synesthetes and controls were reported in the frontal and parietal cortex (whole brain analysis). When restricting the analysis to the visual cortex only a few results (in five studies) were compatible with the involvement of color regions in synesthesia.

### Parietal cortex?

A popular claim is the involvement of parietal regions in synesthesia. Even though parietal cortex is usually not considered as involved in color experience, its role was justified for the binding process involved in synesthetic associations. This was first suggested by Weiss et al. (2005) who observed a strong BOLD signal for graphemes inducing colors in a small group of 9 synesthetes only in the left anterior and caudal IPS (fixed effect analysis). This weak result gained support from two TMS studies (Esterman et al., 2006; Muggleton et al., 2007) that measured a reduction in synesthetic Stroop interference when TMS was applied over the posterior parietal cortex. The weak effect of these underpowered studies (two and five synesthetes) was, however, obtained only on the right side. Only two other fMRI studies, which contrasted synesthetes and controls, measured significant activation for synesthetic stimuli in the left parietal cortex (Rouw and Scholte, 2010; Neufeld et al., 2012a). The peak coordinates of the clusters among the three fMRI studies were all at least 2 cm apart (**Table 2**) and the activations lacked specificity: Neufeld et al. (2012a) contrasted tones against the implicit baseline and for Rouw and Scholte (2010), visual stimuli were not matched between the 42 synesthetes and 19 controls tested. Moreover, activations in nearby regions (though mostly on the right side) were reported for contrasts not directly related to the experience of synesthetic colors (see **Table 2**: van Leeuwen et al., 2010; Sinke et al., 2012). Finally, it is not clear to what aspect of the synesthetic experience these correlates may relate. Parietal areas and parieto-frontal networks are known to be involved in many tasks involving attention. Since the conscious experience of synesthetic colors does require paying attention to the stimulus (Chiou and Rich, 2014, for a review), the specificity of these activations may be difficult to disentangle from attention effects.

### The problem of “control” stimuli

In order to isolate the neural correlates of synesthetic colors, all but two (adaptation) studies had to contrast the responses of inducing stimuli to non-inducing stimuli. Stronger activation was expected for inducing stimuli only in synesthetes, due to the additional synesthetic experience. Hubbard et al. (2005a) and Hupé et al. (2012b) measured such an effect in synesthetes in retinotopic areas. However, Hubbard et al. (2005a) had a similar (though weaker) result for control subjects (Hupé et al., 2012b, did not test control subjects), while other studies (van Leeuwen et al., 2010; Tomson et al., 2013) observed stronger activation to false fonts in both synesthetes and controls, interpreted as related to stimulus novelty.

We should insist on the difficulty of comparing synesthetes and controls, especially when using synesthetic material. Synesthetes may pay more attention to stimuli inducing synesthetic experience, which are much more interesting to synesthetes than controls. Synesthetic associations also involve some emotional content – many synesthetes report enjoying experiencing the synesthetic color of letters, and they report pleasure or disgust when seeing letters whose synesthetic color they particularly like or dislike. This additional emotional reaction, which in a sense belongs to the synesthetic experience, may not only involve the emotional network, but also induce emotion-related physiological responses. Modifications in heart rate and respiratory rate strongly influence the BOLD signal rooted to hemodynamic variations. Moreover, physiological modifications such as blinks have widespread effects not homogenous over the brain (Hupé et al., 2012a). These variables can be measured in the scanner but were never included as cofactors in the reported group comparison analyses. These possible nuisance factors make it problematic to derive inferences from widespread differences observed between synesthetes and controls in regions *a priori* not related to the synesthetic experience, like in the study by Rouw and Scholte (2010), as long as these differences are not replicated.

Retinotopic areas (V1 to V4) are involved in the processing of low- to mid-level visual features. The stronger activation observed across these areas for inducing letters and colored stimuli is unlikely to correspond to the neural correlates of the experience of synesthetic or real colors. On one hand, the visual expertise required for color constancy mechanisms (Bartels and Zeki, 2000) may well be achieved in the ventral stream beyond V4 (Brouwer and Heeger, 2009; Conway and Tsao, 2009; Hupé et al., 2012b,c), as well as the visual expertise for reading letters (Dehaene and Cohen, 2011). On the other hand, visual attention strongly modulates the BOLD signal within retinotopic areas (e.g., Silver et al., 2007; Watanabe et al., 2011), making any comparison of activation for different stimuli difficult to interpret.

### Individual variability between synesthetes

Synesthetes do not all describe their synesthetic experience the same way. Individual differences are certainly an important source of variability that could explain the absence of any observed correlate of synesthetic colors, especially in underpowered studies. Measuring individual differences could help overcome the ambiguity of contrasting synesthetic stimuli to not-so-comparable stimuli. Subjective reports were classified by Flournoy (1893) as belonging to six possible categories, along a strength scale, from hallucination-like experiences and projections to mental images and thought or felt (or even negative, like “5 cannot be red”) experiences. Using questionnaires, several studies classified synesthetes as either “projectors” or “associators” or used a projector/associator scale. The phenomenological quality of synesthetic associations is, however, difficult to capture with such a questionnaire, which does not always correlate with objective measures like the strength of synesthetic priming effects (Gebuis et al., 2009), and which often produces variable responses when synesthetes are retested (Edquist et al., 2006; Hupé et al., 2012b). Objective measures were also proposed to try to capture the different strengths of synesthetic association. Hubbard et al. (2005a) measured the performance of synesthetes in a visual search task, where synesthetic colors should help finding the target (Ramachandran and Hubbard, 2001). Projector-like synesthetes (based on subjective reports) seemed to have higher performances than associators (in fact, several studies have failed to find that synesthetes performed really better than controls on such a task, e.g., Edquist et al., 2006; Rothen and Meier, 2009, or that this task could differentiate synesthetes: Ward et al., 2010; Hupé, 2012; Rich and Karstoft, 2013; see Chiou and Rich, 2014 for a review). Others measured interferences in Stroop-type tests (Hupé et al., 2012b; Rich and Karstoft, 2013; Ruiz and Hupé, 2015).

Several fMRI studies took individual variability into account to qualify their results. For example, Sperling et al. (2006) had results possibly compatible with the involvement of V4 in synesthesia in only 2/4 synesthetes. They commented that this could be due to differences in phenomenology. These two synesthetes reported perceiving a completely colored screen in their mind’s eye when seeing an inducing letter, while the mind’s eye colored image was much smaller for the two other synesthetes (this contingency is of course anecdotal given the low number of subjects). However, Rouw and Scholte (2007) did not find any significant correlation between the score to the Projector/Associator questionnaire and the BOLD signal in any region where they had observed a stronger response for synesthetes than controls (see their Table S3: positive and negative trends across the 18 synesthetes, all *p* > 0.3, one-tailed tests). In their 2010 study on their larger group of 42 synesthetes, Rouw and Scholte did not report the result of the correlations in “significant regions,” unfortunately. However, when categorizing synesthetes as projectors or associators based on the questionnaire, they did not observe any difference between the groups in these regions. van Leeuwen et al. (2010) performed a similar comparison using the same questionnaire (19 synesthetes). They also found no difference between groups in color regions. Hubbard et al. (2005a) did not observe any correlation between the BOLD responses in V4 and performance in the visual search task in their small sample of six synesthetes (*p* = 0.82, our computation; their claimed “brain-behavior correlations” referred to the results of a crowding task; please refer to the Appendix for a thorough discussion of their study). Hupé et al. (2012b) even observed negative trends between the BOLD signals in retinotopic and color areas and performance on the synesthetic Stroop task (nine synesthetes). These two results disqualify the differences between letters and false fonts observed in retinotopic areas as possibly due to synesthetic colors.

Taking individual variability into account has so far not helped in revealing the neural bases of synesthetic colors. These negative results are, however, not conclusive as long as we are not sure that individual variability is correctly captured by either questionnaires or psychophysics tests.

## PART 2. STRUCTURAL CORRELATES OF SYNESTHESIA

The main question is whether synesthetic experience comes from structural brain alterations. Structural alterations were explored using MR imaging in two ways: (1) *Structural morphometry* to investigate whether synesthesia would be associated with local changes of brain tissue volume [gray matter (GM) and white matter (WM)] or fractional anisotropy (FA) modifications and (2) *Connectivity* to explore whether synesthesia would be associated with abnormal connections between specific areas. If correlations were found between synesthesia and structural changes, it would remain to be evaluated whether these changes could explain the experience of synesthesia.

### 2.1. IS THE EXPERIENCE OF SYNESTHESIA RELATED TO STRUCTURAL CHANGES?

We identified eleven studies (**Table 3**). Two MR modalities were used for searching for structural differences between synesthetes and controls: diffusion tensor imaging (DTI, seven studies) for probabilistic fiber tractography and FA analysis, and T1-weighted MR imaging (eight studies) for voxel-based morphometry (VBM) analysis or cortical surface analysis, to identify atrophy or presence of tissue expansion (GM and WM) between groups of subjects (four studies explored both). The synesthetic experience was color in all studies. It was elicited by graphemes (Rouw and Scholte, 2007, 2010; Jäncke et al., 2009; Weiss and Fink, 2009; Hupé et al., 2012b; Melero et al., 2013; O’Hanlon et al., 2013; Whitaker et al., 2014), by tones (Hänggi et al., 2008, one “multiple” synesthete who had also interval-taste synesthesia), by tones or graphemes (Banissy et al., 2012), or by tones or music (Zamm et al., 2013). One important question is, therefore, whether structural changes may affect the function of color centers.

**Table 3:** Structural correlates of synesthesia, morphometry.

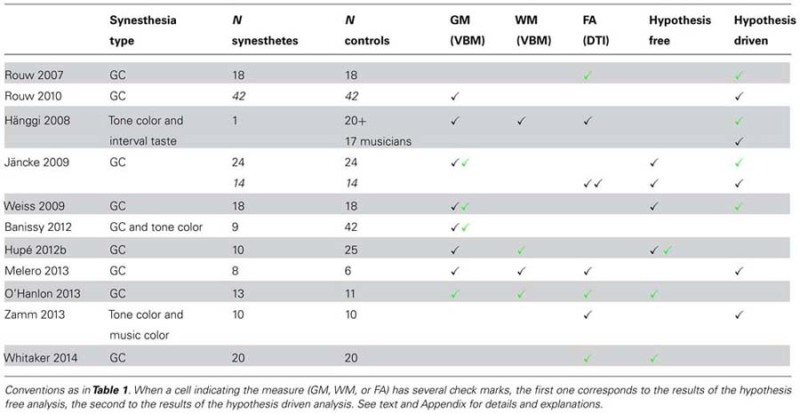

#### Hypothesis free analysis (six studies: five VBM studies, three DTI studies)

Five studies used VBM and computed statistics corrected at the whole-brain level^2^ (Jäncke et al., 2009; Weiss and Fink, 2009; Banissy et al., 2012; Hupé et al., 2012b; O’Hanlon et al., 2013). Three of them (Jäncke et al., 2009; Weiss and Fink, 2009; Hupé et al., 2012b) included a whole brain measure as a covariate. In the other two studies (Banissy et al., 2012; O’Hanlon et al., 2013), differences could be due to a combination of local and global modifications (local GM volume and global brain volume).

No significant GM differences were found in synesthetes compared to controls in four studies [but Jäncke et al. (2009) as well as, apparently, Weiss and Fink (2009), did not test whether controls could have larger values]. Group sizes (synesthetes/controls) were 24/24 (Jäncke et al., 2009), 18/18 (Weiss and Fink, 2009), 10/25 (Hupé et al., 2012b), and 9/42 (Banissy et al., 2012). Only O’Hanlon et al. (2013; 13 synesthetes and 11 controls) reported larger GM values in synesthetes in nine regions.

Although both gray and WM volumes can be assessed using VBM, few explored WM differences for grapheme-color synesthesia, probably because DTI imaging, with adequate computation, is presently more adapted than T1-weighted imaging. Hupé et al. (2012b) reported WM increase in the retrosplenial cortex (bilaterally) and in the left superior temporal sulcus (STS). O’Hanlon et al. (2013) reported larger WM values in synesthetes in six regions.

Three studies used DTI and computed statistics on FA corrected at the whole-brain level (Jäncke et al., 2009; O’Hanlon et al., 2013; Whitaker et al., 2014). Jäncke et al. (2009; 14 synesthetes and 14 controls) found no difference; O’Hanlon et al. (2013; 13 synesthetes and 11 controls) observed increased FA in 14 regions, and Whitaker et al. (2014; 20 synesthetes and 20 controls) found only regions with lower FA.

#### Conclusion of the hypothesis free analysis

Only two studies found large differences between synesthetes and controls. O’Hanlon et al. (2013) observed larger GM, WM and FA values in synesthetes, while Whitaker et al. (2014) observed only lower FA in synesthetes. The widespread differences as well as the lack of consistency of the results suggest that these results are false positives due to methodological issues. Indeed, both studies used inadequate statistical models (please refer to Appendix, Structural studies: Structural morphometry studies). Moreover, they did not include covariates, the study by O’Hanlon et al. (2013) was underpowered, and Whitaker et al. (2014) measured DTI images along six directions only. Hupé et al. (2012b) reported just significant WM differences in two small regions. Their study was, however, underpowered (10 synesthetes only). Therefore, there is no strong evidence so far of any structural difference between synesthetes and controls, in particular for GM, and no observed difference around color centers. WM differences have been hardly (correctly) studied at this level yet.

#### Hypothesis driven or exploratory analysis (eight studies: five VBM studies, four DTI studies, one single-case study)

Similarly to functional results, the absence of significant difference is of course not the proof of the absence of difference, especially when considering the small number of studies and their low power (in particular for WM studies, either using VBM or DTI).

Five VBM studies (three already included in the previous section) explored GM differences in the fusiform gyrus and in the caudal IPS. They all reported differences between synesthetes and controls, either in the fusiform gyrus or the left caudal intraparietal cortex, or both. However, in all of them, these differences were small and not the largest measured, among many possible false positive differences. There were also key methodological differences making the comparison of results difficult. Two studies did not include brain size as a cofactor (Rouw and Scholte, 2010; Banissy et al., 2012). Two studies were underpowered (Banissy et al., 2012; Melero et al., 2013). Only two studies (Jäncke et al., 2009; Weiss and Fink, 2009) used a comparable and sound methodology with reasonably sized groups (of 24 and 18 subjects respectively), while Rouw and Scholte (2010) tested the largest groups (of 42 subjects) to date. The results in the *left* fusiform gyrus were not consistent (increase observed by Jäncke et al., 2009, decrease by Rouw and Scholte, 2010, no difference for Weiss and Fink, 2009). Both Weiss and Fink (2009) and Rouw and Scholte (2010) reported differences in the left caudal IPS. However, Weiss and Fink (2009) identified a small cluster, presumably in hIP3 (human intraparietal area 3), while Rouw and Scholte (2010) measured GM difference in hIP2. Moreover, this difference was part of a large cluster (almost 3 cm^3^) of larger GM in synesthetes, whose center of gravity was 2 cm away from the coordinates reported by Weiss and Fink (2009), suggesting the absence of overlap (**Table 2**; note that even if there was some overlap it may not be conclusive because Rouw and Scholte computed cluster extent statistics that do not allow any inference about where the difference is within this large cluster). Jäncke et al. (2009) and Weiss and Fink (2009) both measured larger GM in the *right* fusiform gyrus, using similar methodology. However, the small cluster reported by Weiss and Fink (2009) was more posterior and at least 2 cm away from the clusters reported by Jäncke et al. (2009). Finally, both Banissy et al. (2012; nine synesthetes vs. 42 controls) and Melero et al. (2013; eight synesthetes vs. six controls) did not observe any difference in the right fusiform and left IPS ROIs defined by Weiss and Fink (2009). There is, therefore, no consistency across the results of these five studies.

Only Melero et al. (2013) explored WM difference at a lenient, exploratory threshold. Their study was underpowered and the analysis suboptimal. However, it is worth noting that they measured no difference in the fusiform gyrus or in the IPS, as well as no difference in the retrosplenial cortex and the STS, where Hupé et al. (2012b) had observed higher local WM volume after family-wise error (FWE) correction.

Four DTI studies (two already included in the previous section) explored FA differences using liberal thresholds or *a priori* hypotheses. Rouw and Scholte (2007) identified four possible regions (*p* < 0.0025, minimal extent = 40 mm^3^) with larger FA in a group of 18 synesthetes (compared to 18 controls), one of them in the right inferior temporal cortex (the closest GM difference, 7.5 mm away, was one of the clusters reported by Jäncke et al., 2009). With a similar methodology, however, Jäncke et al. (2009) did not replicate this result on groups of 14 subjects, even at a much more liberal threshold (*p* < 0.01, minimal extent 30 mm^3^). With a weaker methodology and small groups, Melero et al. (2013) also did not replicate this result at a lenient threshold. Zamm et al. (2013) compared 10 music-color synesthetes to 10 controls. They measured FA only in WM pathways that pass through both temporal and occipital regions. They did not find any difference in the ILF (inferior longitudinal fasciculus), which connects the occipital and temporal cortices. Rouw and Scholte (2007) had indeed observed that their higher FA observed in the right inferior temporal cortex of synesthetes was close to the ILF. Zamm et al. (2013) reported a slight increase in the right IFOF (inferior frontal-occipital fasciculus), which connects the occipital and frontal cortices.

In a thorough single-case study, Hänggi et al. (2008) compared a single subject who had synesthetic experiences triggered by tones or music intervals to controls, measuring WM and GM with VBM, as well as FA with DTI. Since this synesthete was a professional musician and had also perfect pitch, they had a control group of professional musicians, some of them with perfect pitch. They did not try to correct for multiple comparisons but a consistent pattern of results emerged across all their analyses. This synesthete had larger values of WM and FA in areas involved in the processing of the inducing stimuli (auditory area). However, these differences could be related to absolute pitch rather than synesthesia since the critical comparison with musicians with absolute pitch was not conclusive.

#### Conclusion of the hypothesis driven or exploratory analysis

Using liberal statistical thresholds or *a priori* hypotheses, there was no consistent result across studies. Therefore, there is no evidence so far of any structural difference between synesthetes and controls, in particular in regions supposed to code the synesthetic experience (color). The empirical data in favor of the Null hypothesis is, however, weak, except for GM differences. WM has been studied with VBM in only three group studies, and not appropriately (methodological issues, or too small samples, or both). Seven studies used DTI, but three of them made questionable choices for their analysis; one was a single-subject study. Among the remaining three studies, only one reported results corrected for multiple comparisons (but without indicating the procedure). In addition, the accuracy of FA maps depends on the DTI acquisition scheme. The lower the number of directions, the noisier the estimations at the voxel level (Ni et al., 2006; Giannelli et al., 2009). The studies reported here used different but overall small numbers of directions (< = 32). The study of possible WM differences in synesthesia using structural morphometry is, therefore, still in its infancy.

### 2.2. IS THE EXPERIENCE OF SYNESTHESIA RELATED TO CONNECTIVITY CHANGES?

Eight studies assessed connectivity changes (**Table 4**). Brain connectivity was used to compare synesthetes vs. controls with structural MRI (one study), functional MRI (four studies), and EEG recordings (two studies). One additional fMRI study compared different models in a population of synesthetes only. Synesthetes were the grapheme-color type in all but one study (auditory-visual synesthetes, also inducing colors). Most studies were interested in discovering whether synesthesia was either due to connectivity changes between the regions (of the fusiform gyrus for grapheme-color synesthesia) coding the inducer and the concurrent (cross-activation theory), or due to a difference of neuronal transmission (disinhibited feedback theory), possibly more widespread.

**Table 4:** Structural correlates of synesthesia, connectivity.

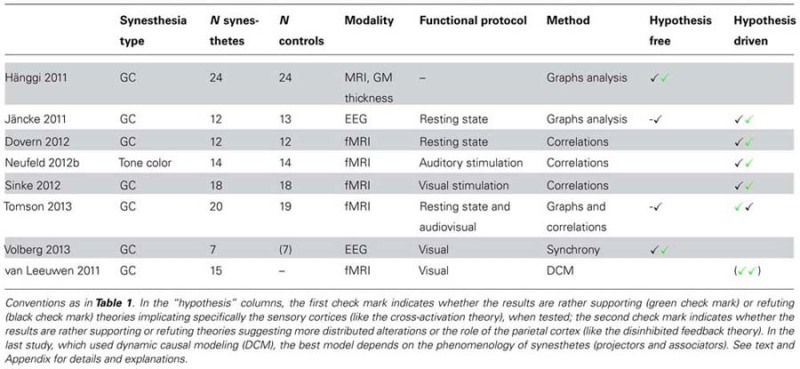

#### Hypothesis free analysis (four studies)

Four studies targeted brain connectivity using very different signals (structural MRI, EEG, and fMRI). By analyzing cortical thickness, Hänggi et al. (2011) observed differences of connectivity between 24 synesthetes and 24 controls. Synesthetes had reduced small-world architecture, corresponding to hyperconnectivity at the local level, though not within the fusiform gyrus as predicted by the cross-activation theory. Jäncke and Langer (2011) measured functional signals with EEG, during rest. They computed correlations within the source space of electrical signals and for different frequency bands. Contrary to Hänggi et al. (2011), they did not find any general difference in global connectivity between 12 synesthetes and 13 controls. Using fMRI during resting state and audio or audiovisual stimulation, Tomson et al. (2013) also did not find any difference of global network metrics between 20 synesthetes and 19 controls. Volberg et al. (2013) measured both local and distant synchrony between EEG electrodes after the presentation of inducing and non-inducing graphemes in seven synesthetes. They obtained results in favor of the disinhibited feedback theory of synesthesia, in particular a decreased long-range coupling within the theta range starting at 280 ms, compatible with a decrease of inhibition for inducing letters in synesthetes: this theta decrease was followed indeed at 400 ms by an increase of local synchrony in the beta band, supposedly involving the left fusiform gyrus.

#### Conclusion of the hypothesis free analysis

Overall, no consistent difference in functional connectivity was observed between synesthetes and controls. The major reported differences were based on the indirect measure of local cortical thickness (Hänggi et al., 2011). It is not clear what can be inferred from covariations in cortical thickness, and what exact relation they have with functional connectivity. Moreover, such connectivity measures require that they are not biased by thickness differences between synesthetes and controls. Many local differences were, however, present between both groups that may have biased all the statistics of connectivity to an unknown degree. Results based on the analysis of phase coherence of EEG signals during stimulation favored the disinhibited feedback theory of synesthesia (Volberg et al., 2013). The results were, however, not reliable because of the low number of subjects and the absence of systematic comparison with the control group. Both these results and those by Hänggi et al. (2011) were explicitly not compatible with the cross-activation theory of synesthesia.

#### Hypothesis driven analysis (six studies, including two studies from the previous section)

Jäncke and Langer (2011) measured the degree of functional connectivity between the EEG sources of 84 anatomical areas (12 synesthetes). Given the high number of possible comparisons, they reported uncorrected effects and insisted on regions for which they had *a priori* hypotheses. Synesthetes had higher values than controls in the parietal cortex but not in the fusiform gyrus.

Dovern et al. (2012) performed an independent component analysis (ICA) on resting-state functional MRI data in 12 synesthetes and 12 controls. They analyzed the functional connectivity between seven intrinsic connectivity networks (ICNs) potentially relevant to grapheme-color synesthesia (involving the visual cortex, the auditory cortex, or the intraparietal cortex). They found two connections significantly stronger in synesthetes, between both the medial and lateral visual networks and the fronto-parietal network. They also computed correlations between the BOLD time course in bilateral V4 and each brain voxel. No difference between synesthetes and controls survived correction for multiple comparisons.

Using fMRI, Neufeld et al. (2012b) and Sinke et al. (2012) analyzed functional connectivity in 14 tone-color and 18 grapheme-color synesthetes, respectively (compared to as many controls), during stimulation inducing synesthetic colors. They computed correlations between BOLD time-courses using seed areas in the left inferior parietal cortex (both studies) and the auditory cortex (Neufeld et al., 2012b) or regions of the visual cortex (Sinke et al., 2012). They did not find any stronger functional connectivity between the visual and auditory cortex in tone-color synesthetes, and no increased connectivity in grapheme-color synesthetes when using the seed functionally defined as responding to visual letters. Both results brought evidence against the cross-activation theory as well as against the possible involvement of color areas in color synesthesia. In both studies, there was some evidence in favor of the involvement of the left inferior parietal cortex, bringing support to the disinhibited feedback theory.

Tomson et al. (2013) analyzed fMRI data of 20 synesthetes using a similar ROI based strategy. They did not find any evidence for the involvement of parietal regions, even without any correction for multiple comparisons. They considered some of their results as compatible with increased local connectivity within the visual cortex, in particular between regions potentially coding colors and graphemes. Their results were, therefore, more in favor of the cross-activation theory than the disinhibited feedback theory.

van Leeuwen et al. (2011) used dynamic causal modeling (DCM) to test two predefined models corresponding to both theories in 15 synesthetes, using fMRI. They found no strong preference across all synesthetes for either the bottom–up (cross-activation) or the top–down (disinhibited feedback) model. However, the bottom–up model was better for the 10 projectors of the group while the top–down model was better for the five associators. Among projectors, the six mental screen projectors (also called strong associators) had intermediate preferences between both models.

#### Conclusion of the hypothesis driven analysis

These six studies relied on *a priori* information for the definition of nodes or seeds for connectivity analyses, in order to test the implication of color and parietal (binding) regions in color synesthesia. Four studies found evidence against connectivity changes involving color areas (cross-activation theory), one found some evidence, and one suggested that the cross-activation theory applies only to projector synesthetes (van Leeuwen et al., 2011). The other studies did not try to classify their synesthetes using questionnaires. Since associators are more frequent among grapheme-color synesthetes, the dependence of connectivity on individual differences is compatible with the first four but not the fifth study (Tomson et al., 2013), the only one that was compatible with a role of color centers. All but one (Tomson et al., 2013) study found some evidence in favor of the role of the parietal cortex. However, these studies relied on very different methods to evaluate its role. Critically, they did not use any consistent method to define the seeds or nodes for connectivity analysis. Tested parietal regions were up to several centimeters apart (**Table 2**) and the measured increases of connectivity involved different regions (and signals) across all studies. A similar comment applies to the definition of color regions: the lack of evidence in favor of their role might be due to the inadequate definition of color seeds.

## Discussion (Part 2)

Among 19 studies, we did not find any clear evidence of structural brain alterations in synesthetes, either local differences or differences in connectivity, at least when considering the data with no *a priori*.

### Data heterogeneity

This negative result is based on a very heterogeneous set of data, most studies testing different modalities and performing different analyses, due to the complexity and the absence of standard among all possible analyses. Even for similar studies, for example VBM, the measure was not always comparable among studies: generally, the volume of brain tissues is related to the whole brain volume (larger brains contain larger tissue volume). One way to account for this confound is to use total intracranial volume as cofactor. This was apparently not done and could introduce a bias in some studies (Rouw and Scholte, 2010; Banissy et al., 2012; O’Hanlon et al., 2013). In our study, for example (Hupé et al., 2012b), there was no difference in brain size between the groups of synesthetes and controls (*p* = 0.55). However, when not using brain size as a cofactor, the largest difference in WM measured in the right retrosplenial cortex was just significant (uncorrected *p* = 0.026). With brain size as a cofactor it reached *p* < 10^-10^ and thus could survive the correction for multiple comparisons.

Statistical models used were also different across studies and were not equally optimal. These models were inspired by those used in fMRI, even though the properties of the underlying signals are quite different. The application of the random field theory, already delicate for fMRI, may be even more problematic for structural data, especially for cluster extent statistics. White matter tracts, constructed based on FA (DTI studies), are not random fields. The problem of multiple comparisons may, therefore, be even more difficult to handle for structural than for fMRI studies. For connectivity analyses, the number of possible comparisons is even larger (Jäncke and Langer, 2011), since it may include all possible combinations between tens or 100s of seeds or nodes, which themselves can be defined in many different ways. “Generic” solutions like false discovery rate (FDR) or permutations are not suitable because FDR depends crucially on the definition of the family of tests (which is typically unknown for the present exploratory studies) and not much inference is permitted by permutation tests (beyond the lack of exchangeability) in the absence of other, parametric, information (see Hupé, 2015: third section “Pitfalls of MRI statistics”).

### Brain behavior correlations

We are not able to identify the causal chain between potential structural differences and behavior. Like for genetic association studies, we may need to perform comparisons between groups of hundreds or thousands of subjects to assess whether an observed significant correlation does indeed suggest causality (Ioannidis, 2005a,b). Put simply, observed structural differences between two small groups of synesthetes and controls may be related to random differences that exist between any two groups of people, rather than synesthesia, given the very large number of potential individual differences that may have a morphometric counterpart. As a first obvious difference, recruitment procedures between synesthetes (most self-referred, some of them participated in several studies) and controls were different in most studies reported here. Covariates related, for example, to personality or cognitive abilities could be measured and included as covariates in the analysis (Chun and Hupé, 2013b). Tens of such measures are easily collected, meaning that ten times more subjects should be tested to overcome the curse of dimensionality. This simple thought experiment shows that all structural studies that looked for differences between synesthetes and controls were severely underpowered.

### Hypothesis-guided studies

Most studies presented here used an alternative strategy to get around the problem of multiple variables and comparisons: they tested precise models using additional information. For example, since color was the synesthetic concurrent in all studies, searching structural differences specifically involving color networks was legitimate. The structural results suggesting the involvement of the fusiform gyrus were not consistent and most connectivity results argued against its role. However, these analyses faced the major problem of identification of “color networks” in individual subjects. Most studies used ROI based on functional localizers (sometimes poorly related to color processing itself) or on coordinates from the literature, with no guaranty that the most relevant brain tissues were compared between synesthetes and controls. More structural results exist in favor of the role of the parietal cortex in synesthesia. However, the *a priori* hypothesis concerning its functional role in synesthesia is not grounded (see Part I). The structural evidence was, in fact, poorly characterized and there was no consistency across studies about the precise anatomical location of which part of the parietal cortex was supposed to be involved. The lack of any clear-cut result about the functional correlates of synesthetic colors (see Part I) makes the structural comparisons between synesthetes and controls (both positive and negative results) based on such results inconclusive.

### Individual variability between synesthetes

With or without *a priori* information, if correlations were found between synesthesia and structural changes, it would remain to be evaluated whether these changes could explain the experience of synesthesia. Similarly to functional results, the measure of individual differences about the experience of synesthesia is a powerful tool to test whether potential differences are related to synesthesia. Only few structural studies measured individual differences and computed related correlations in the regions where they had found potential differences from controls. Rouw and Scholte (2007) measured a positive correlation between FA in the right inferior temporal cortex (where FA was larger in synesthetes than controls) of 18 synesthetes and scores on the Projector/Associator questionnaire. The result, however, was statistically weak and the computation of the score questionable (see Appendix, Structural studies: Structural morphometry studies). Weiss and Fink (2009) did not find any correlation between the projector/associator scale and local GM volume in the fusiform gyrus or the IPS of 18 synesthetes. Hupé et al. (2012b) observed no correlation between local WM volume in the retrosplenial cortex and the strength of color associations measured in nine synesthetes with Stroop-like tasks. Zamm et al. (2013) measured a significant correlation between FA in the right IFOF and the consistency of synesthetic associations of 10 synesthetes. Dovern et al. (2012) also reported a correlation between the consistency scores of 12 synesthetes and the connection strength of the lateral visual ICN with the auditory ICN. In both cases, the correlation was, however, statistically weak (few subjects, multiple comparisons), and the measure poorly differentiated synesthetes from controls. van Leeuwen et al. (2011) measured a correlation between the projector/associator scale of 15 synesthetes and preference for either the top–down or the bottom–up model. There was, however, no comparison with a control group to assess whether any of these models was related to synesthesia.

Similarly to functional results, taking individual variability into account is promising but did not reveal consistent results across studies. Different measures were computed. An objective, consensual measure of individual variability is still lacking. There is no evidence so far that the experience of synesthesia comes from structural brain alterations.

## CONCLUSION

We did not find any clear-cut empirical evidence so far about the neural correlates of the subjective experience of synesthesia. We did not find any structural or functional anomaly in the brain of synesthetes that could explain synesthesia. In our view, most published studies to date show, in fact, that the brains of synesthetes are functionally and structurally similar to the brains of non-synesthetes.

### MRI RESEARCH IN COGNITIVE NEUROSCIENCE: THE EXAMPLE OF SYNESTHESIA

Yet, most published synesthesia papers described here claimed to have found neural correlates of synesthesia. Almost all these claims were unsupported due to statistical errors, questionable methodological choices, or low statistical power. We described these problems in (Hupé, 2015). We reported detailed explanations for each study in the Appendix. These problems are not specific to synesthesia research (e.g., Celle et al., 2015), and several studies have warned that misuse of statistical inference based on null hypothesis significance tests (NHSTs), low power, publication bias and pressure for rapid publication, are endemic to psychology and neuroscience (e.g., Meehl, 1967; Ioannidis, 2005b; Button et al., 2013; Ioannidis et al., 2014). These problems are aggravated in cognitive neuroscience using MRI because of the cost of the experiments and the complexity of image processing pipelines and statistical analysis procedures. As long as the neural code of mental states and cognitive operations has not been cracked, cognitive neuroscience is fated to look for correlations between cognitive processes, which are often difficult to control properly, and indirect measures of brain activity or structural features produced by brain imaging techniques. The control of the false positive risk across multiple comparisons is an ill-posed problem within the context of NHST. Twenty years ago, an elegant solution for the analysis of PET and fMRI images was proposed based on the random field theory. However, this theory provides only an approximate solution and, to be valid, requires that many conditions be met (Nichols and Hayasaka, 2003). These conditions are difficult to verify and seem to have sometimes been forgotten with the accessibility of end-user software. For structural and functional connectivity analysis, we observed an even wider diversity of analysis pipelines (Carp, 2012), with almost no two studies using the same procedure. We came across very ingenious methods using powerful mathematical tools, but we found no gold standard. Group analyses of individual brains, which are inherently different, are, in any case, problematic. We are not sure that the current tools allow us to correctly study subtle brain mechanisms, such as those involved in synesthesia, in analyses based on a reasonable (low) number of subjects.

### THE NEURAL BASES OF SYNESTHESIA: ALTERNATIVE MODELS

The majority of published studies focalized on specific brain areas. Indeed, for grapheme-color synesthesia, activation of color centers due to cross-activation or feedback (due to either functional or structural differences between synesthetes and controls) seemed the only logical possibility, which was already proposed in the XIXth century (review by Suarez de Mendoza, 1890). As long as no other mechanism is proposed, methodological critiques of “positive” results in favor of such a hypothesis may remain unconvincing. Alternative propositions involve global changes within distributed cortical networks, but this emergent field has not yet reached maturity, both in terms of validation of the methodology and theoretical interpretation.

Synesthesia is often described as a neurological condition: the cause of synesthesia would be a structural or functional anomaly in the brain of synesthetes. Findings of functional or structural differences in synesthetes have often been interpreted to support such a view. Note, however, that functional results do not necessarily speak to whether synesthesia is a neurological condition or not. Synesthetes do have a different subjective experience than non-synesthetes when confronted with their idiosyncratic synesthetic material, a different subjective experience that must be reflected in the brain (where else?). The question at stake is whether we possess the methodological and theoretical tools to observe it. In any case, if none of the proposed structural or functional differences should be confirmed, this would speak against synesthesia being a neurological condition. But, then, what could be the nature of synesthesia?

In the early 2000s, the neurological hypothesis was often contrasted against a memory hypothesis. Chiou and Rich (2014) argued, indeed, that the experience of synesthetic colors more closely resembles color memory than color perception. Since synesthetic associations appear during childhood, they may just be a special kind of childhood memory – special because these memories are deprived from their autobiographical context. But in that case, one should be able to trace back the origin of these souvenirs. For grapheme-color synesthesia, usual suspects were colored alphabets and toys (Calkins, 1893), widely used since the XIXth century when grapheme-color synesthesia was first described. In an ambitious endeavor, Rich et al. (2005) collected 136 alphabet books that a sample of 150 synesthetes could have had access to during their childhood. Most of these books did not use color, and synesthetic colors matched those of one of the alphabets for only one synesthete. Such a result seemed to rule out the memory hypothesis. However, Witthoft and Winawer (2006) reported a single case where the color magnets present during the childhood of a synesthete did match the synesthetic colors. They later managed to discover ten other similar examples (Witthoft and Winawer, 2013). Such evidence shows that the “choice” of colored associations could be triggered by the child’s environment. The question, then, is why this is not the case for the majority of synesthetes. The explanation could lie in the creative mind of children. Ward and Simner (2003) had detailed the precise phonemic associations of a lexical-gustatory synesthete. They showed that the taste of words was influenced by very fine-grained phonemic properties, like allophony. However, they could trace back the origin of many associations between phonemes and tastes to food words (see also Simner and Haywood, 2009), not necessarily via direct semantic links (“bar” tasted like milk chocolate) but also via more complex links, for example lexical-phonological (“Virginia” tasted like vinegar). By such recursive phonemic or semantic associations, the final repertory of word-taste associations lost its obvious link to food words and looked arbitrary. Grapheme-color synesthetes seem to experience as well a period of progressive construction of associations (Simner et al., 2009; Simner and Bain, 2013). Children may pick up color choices from the different sources that they may encounter over time (colored alphabets, colored toys with letters, colored printed material, etc...), and modify them over time until they eventually stabilize, at least to a certain degree (Simner, 2012). According to this scenario, in most cases, we should not find a single origin of the specific associations between graphemes and colors. Similar explanations were proposed for sequence-space synesthesia (Price and Pearson, 2013), which could result from “extensively rehearsed associations, established for example via normal processes of visuospatial imagery” (Price and Mattingley, 2013). If synesthetic associations are memories of a special kind, the neural correlates of synesthesia may be difficult to identify as long as detecting the signature of memory contents in the brain is out of reach.

## Conflict of Interest Statement

The authors declare that the research was conducted in the absence of any commercial or financial relationships that could be construed as a potential conflict of interest.

## Footnotes

1 We also considered that cluster extent statistics, though widely used, may not fully control the risk of false positives and may face an overfitting issue. See Hupé (2015): third section “Pitfalls of MRI statistics.”

2 Other whole brain, hypothesis-free analyses that did not report statistics corrected for family-wise error (FWE) are considered as exploratory, and are treated together with hypothesis driven analyses.

## APPENDIX

Critical summary of the studies presented in this review

FOREWORD

FUNCTIONAL STUDIES

fMRI (and one pet) studies, (grapheme)-color synesthesia
  Group studies, auditory stimuli
  Group studies, visual stimuli
  Single-case studies
fMRI studies, experiments on other types of synesthesia
EEG, MEG
Other studies

STRUCTURAL STUDIES

Structural morphometry studies
  Group studies
  Single-case study

Connectivity studies
  Synesthetes vs. Controls
  Individual differences among synesthetes

Studies are listed by increasing publication year within each section.

### FOREWORD

All studies reviewed here based their conclusions on Null Hypothesis Significance Tests (NHST). In a companion paper (Hupé, 2015) we explained what we understood of NHST so readers can evaluate precisely on what basis we drew interpretations of published results and they can decide for themselves to follow us or not when we reached conclusions different from those made by the authors. MRI statistics also involve much more complex issues than standard statistical inference. Analysis pipelines vary a lot among studies, even for those using the same software, and there is no consensus which pipeline is the best. Algorithms and software also evolved a lot over the past 20 years. We did our best to retrieve a few key parameters used in each study to assess discrepant results: contrasts used, voxel or cluster statistics, threshold for cluster statistics, data smoothing, theory or permutation-based inferences (for explanations, see Hupé, 2015: third section “Pitfalls of MRI Statistics”).

The following “common mistakes with statistical inference” were described by Hupé (2015) in the second section, paragraphs 1–6:

[1, Sample size]

[2, Accepting the Null]

[3, Double dipping]

[4, A hypothesis is not an *a priori*]

[5, Random vs. Fixed effect]

[6, Selective reporting]

### FUNCTIONAL STUDIES

#### fMRI (AND ONE PET) STUDIES, (GRAPHEME)-COLOR SYNESTHESIA

(Synesthetes were the grapheme–color type unless specified. Color was the concurrent synesthetic experience in all studies).

##### Group studies, auditory stimuli

***Paulesu 1995: six synesthetes and six controls, voxelwise peak statistics.*** Using PET, Paulesu et al. (1995) measured differences of signal for auditory words and pure tones in six synesthetes (who would be described nowadays as “grapheme–color,” since the authors observed that the same phonemes corresponding to different letters were associated to different colors) and six controls ([1, Sample size]). They observed more significant differences (activations and deactivations when comparing words and tones) in synesthetes than in controls ([2, Accepting the Null]), correcting for multiple comparisons based on random field theory (peak heights of *t*-statistics were considered as significant for corresponding *z*-score >3.7). They indicated that at the Talairach coordinate of putative V4 (Zeki et al., 1991) the signal was stronger for words than tones in synesthetes, but this difference did not reach even a weak statistical threshold (*z* = 2.1, corresponding to uncorrected *p* = 0.05). They did not report the result in the control population but the interaction between group and stimulus was not significant, even when applying no correction for multiple comparisons. The authors reported that the differences of activation to words (compared to tones) observed between synesthetes and controls were still significant when computing the interaction between stimuli and group, but this result was obtained without correcting for multiple comparisons (their threshold was *z* = 2.4, corresponding to uncorrected *p* = 0.01). They justified such a lenient threshold by arguing that such analysis was “hypothesis driven” by the observations made within each group, making a circularity error ([3, Double dipping]). The interactions reported in their tables had *z* scores below 3.1 (except the right insula, *z* = 3.5), meaning that when correcting for multiple comparisons there was no difference between synesthetes and controls.

Our conclusion: if applying a stringent measure to control the risk of false positives, there was no significant difference between synesthetes and controls.

***Nunn 2002: 10 synesthetes and 10 controls, cluster extent statistics.*** Nunn et al. (2002) used a paradigm similar to Paulesu et al. (1995) with fMRI (1.5T scanner). They asked subjects to listen passively with eyes closed to auditory words vs. tones, presented for 5 min within 30 s blocks. In their first experiment, they compared the activation maps (group median of the modulation by words) for 10 grapheme–color synesthetes and 10 controls (cluster extent statistics; voxel threshold was indicated in the Methods *p* = 0.05, but the legends indicated a more likely *p* = 0.0005; such threshold was computed by comparing the group median to the distribution of medians obtained with permutation of the time series within each subject. The minimum cluster size was 4 to reach pFWE = 0.05, computed on the basis of empirical measurements obtained at rest. No spatial smoothing was indicated.). In their second experiment they performed a similar comparison between six synesthetes (three of them were retested) and eight controls, but at a higher (less conservative) statistical threshold. They observed activation in the left infero-temporal cortex for synesthetes only, which they supposed lay in the V4/V8 region (but no accurate delineation based on fMRI retinotopic mapping was performed). However, they observed less activation overall in controls than in synesthetes (so the differences between groups may not be related to the synesthetic experience *per se*), and, critically, they did not report group comparisons.

They also measured the responses to colored Mondrians for the six synesthetes and eight controls of the second experiment, in order to test whether real and synesthetic colors activated the same brain regions. On average, they did not observe any significant color activation on the left side for synesthetes (where they had observed larger activation for words compared to tones), and therefore did not find any overlap of activation for colored Mondrians and heard words triggering synesthetic colors. They also performed a clever control task, where they trained 10 control subjects to associate colors to words and then scanned them. The authors insisted on the contrast between two conditions, where subjects had either to remember or to imagine the color, and they reported no difference for these two conditions in the left V4/V8 region of interest (ROI) defined based on their previous experiment (10 voxels overlap between the V4/V8 ROI in synesthetes for words–tones and the colored region reported in the literature). As pointed by Blake et al. (2005), it is not sure that the different instructions really induced a different imaging strategy in subjects. Nunn et al. (2002) also contrasted words against tones across both conditions and computed cluster statistics within an area of 10 mm surrounding the 10-voxels V4/V8 ROI. They did observe significant cluster(s) when thresholding voxels at *p* < 0.05, but they insisted that there was only one overlapping voxel with the 10-voxel ROI, which they dismissed as a likely false-positive. They did not show the brain map for the contrast of words minus tones, but reported other brain activation this time for cluster extent statistics when thresholding voxels at *p* = 0.001.

The critical between-group comparison for the words-tones contrast, between synesthetes and controls who had learned the associations, was not provided.

Our conclusion: they did not report between groups effects ([6, Selective reporting]) and therefore did not report any statistical difference between synesthetes and controls. The authors concluded otherwise by comparing statistical maps ([2, Accepting the Null]). They did not observe any common activation by real and synesthetic colors.

***Gray 2006: 8 + 6 synesthetes and five controls, cluster extent statistics.*** A follow-up study was performed by Gray et al. (2006), with the same procedure and probably with the same 1.5T MRI machine (no indication was provided), and apparently synesthetes already tested in the study by Nunn et al. (2002) but two new ones (see Table 2 by Simner et al., 2014). They computed contrast maps (words–tones) for three groups of subjects: eight ACE (“Alien Color Effect”) synesthetes (color words like “blue” are experienced with a different color, usually driven by the first letter of the word), six non-ACE synesthetes and five controls ([1, Sample size]). For both groups of synesthetes but not controls ([2, Accepting the Null]), they observed significant activations in the ventrolateral region of the left temporal cortex [cluster extent statistics like in Nunn et al. (2002) this time thresholding voxels at *p* = 0.0375; minimum cluster extent was not reported; cluster-size threshold was *p* = 0.001, indicated to correspond to a threshold where “less than one false positive cluster was expected” over the whole brain]. The group comparison between both groups of synesthetes revealed no significant difference in this region, but, again, the critical comparison between synesthetes and controls was not reported ([6, Selective reporting]). Moreover the peak activation for words was found this time at the Talairach coordinates corresponding to the Visual Word Form Area, more anterior than in the 2002 study. There was no overlap with the “V4” regions activated by colored Mondrians.

Our conclusion: no reported statistical difference between synesthetes and controls; no common activation by real and synesthetic colors.

***Gaschler-Markefski 2011: six synesthetes and seven controls, ROI statistics.*** Gaschler-Markefski et al. (2011) also used the auditory modality to trigger synesthetic colors in responses to words, in comparison to tones that rarely elicited colors. They compared the BOLD signal of six synesthetes ([1, Sample size]) and seven controls (3T scanner, low noise gradient echo sequence) within a volume restricted to the temporal and occipital lobes. Subjects were instructed to keep their eyes closed, like in the previous experiments, listening to 60 s blocks of tones, words, or silence. They had to press a button after each tone or word. The authors performed ANOVAs on activated voxels in 10 regions of interests (five on each side; the dependent measure was the product of the number of significant voxels by their relative BOLD signal change). When selecting in each ROI the voxels activated by tones or words (in comparison to baseline, *p* < 0.05), they observed no significant condition by group interaction below *p* = 0.05 (uncorrected). When selecting voxels that responded more to words than tones, they observed a difference between synesthetes and controls only in the left inferior temporal gyrus, at *p* = 0.05 using a one-tailed test ([3, Double dipping]: they computed a one-tailed test because they observed a stronger response to words in controls), with a stronger response to words in the control group (they also observed that, over all slices, controls tended to have stronger responses to words than tones, which was not the case in synesthetes). Critically, they did not observe any stronger BOLD signal in response to words (that elicited synesthetic colors) in the visual cortex of synesthetes (occipital lobe and fusiform gyrus).

Our conclusion: no activation in the visual cortex by synesthetic colors, no reliable difference between synesthetes and controls.

***Neufeld 2012a: 14 synesthetes and 14 controls, two-tailed voxelwise peak statistics.*** Neufeld et al. (2012a) tested a group of synesthetes who experienced synesthetic colors (and forms) this time in response to tones. They compared the BOLD responses to different sounds (major, minor and dissonant played by different instruments) using fMRI (1.5T scanner) in 14 synesthetes and 14 controls. The only difference between synesthetes and controls was a stronger activation for synesthetes in the left inferior parietal cortex (IPC; peak heights of F statistics corrected for multiple comparison based on SPM5 random field theory, pFWE < 0.05, peak activations reported if cluster extent > 10; 8 mm FWHM smoothing; additional *t*-tests were performed to reveal the sign of the difference). ROI analysis at the V4 coordinates did not reveal any difference (the statistical threshold was not reported).

Our conclusion is the same as the authors: no activation in the visual cortex by synesthetic colors, even when using *a priori* hypothesis; stronger signal in synesthetes for tones vs. baseline (that is, scanner noise) in the left IPC (41 8-mm^3^ voxels in Brodmann area 40: see **Table 2**).

##### Group studies, visual stimuli

***Weiss 2005: nine synesthetes, one-tailed voxelwise peak statistics.*** Weiss et al. (2005) measured the BOLD signal of nine synesthetes in a 1.5T scanner. This study was influential because it suggested a stronger involvement of the intraparietal cortex than color areas in the experience of synesthesia. They used a block design with the presentation of series of letters chosen for each synesthete to induce either a strong or no synesthetic experience of color. In each block, the set of letters was presented as light gray or with colors inconsistent with the synesthetic colors. The contrast between blocks of synesthetic and non-synesthetic letters, both presented either in gray or in color, allowed them to search for the neural correlates of synesthetic colors. When correcting for multiple comparisons at the level of the whole brain volume (SPM 99, peak height of *t*-statistics, pFWE < 0.05, no minimum extent; 10 mm FWHM smoothing), they observed two significant clusters within the left intraparietal cortex (see **Table 2**). However, this result was obtained only with a fixed-effect model, which does not allow generalization to the population of synesthetes. When performing a random-effect analysis, the two clusters had more than 20 contiguous voxels with *p* < 0.001 (uncorrected). Weiss et al. (2005) also tested whether letters inducing synesthetic colors activated more some voxels than non-inducing letters within a 10-mm ROI centered on the peak activation revealed for real colors, within the fusiform gyrus. While at the peak locations for real colors there was less BOLD activation for synesthetic colors (see their Figures 2A,B), they observed at least 1 voxel more activated by inducing letters 9 mm away on the left side (max *t* = 2.8, pSVC = 0.073).

Our conclusion: no definitive evidence of any activation specific to synesthetic colors. The authors concluded about the role of the parietal cortex, but this is statistically valid only within their tested population ([5, Random vs. Fixed effect]). The careful interpretation of their data is that if there is any specific activation, it is more likely in the left parietal cortex than in the ventral visual cortex. Weiss et al. (2005) also considered that their weak trend observed for synesthetic colors in the left fusiform gyrus was compatible with other reports of activation of the left fusiform gyrus during synesthesia. We would rather consider that, if making the hypothesis that color regions are involved in synesthetic colors processing, voxels most involved in, respectively, color and synesthetic processing do not seem to overlap.

***Hubbard 2005a: six synesthetes and six controls, ROI statistics.*** In a landmark study, Hubbard et al. (2005a) compared fMRI activations in two groups of six synesthetes and six controls (1.5T scanner). They presented characters (letters and numbers) visually and contrasted the responses to false fonts (block design). They did not perform a whole brain analysis but compared the difference of beta weights for characters and false fonts in regions of interests: retinotopic areas V1–V4, as well as in what they called a “grapheme area.” The between-group comparison of the average signals revealed slightly stronger response differences in synesthetes in V1, V2, V3, and V4, not V3A and not in the grapheme area. They indicated that only in V4 this difference reached the significance level (*p* < 0.05), apparently using a one-tailed test (we recomputed *p* = 0.093 with a non-parametric two-tailed Mann–Whitney test; Hupé et al., 2012b). This small difference was statistically not stronger in V4 than in the other visual areas (as evidenced in their Figure 5). Moreover any “significance” would disappear ([1, Sample size]) if removing for example subject JAC (as a cross-validation procedure; moreover, Brang et al. (2010) reported that for this synesthete “particular characters in the false fonts […] began to appear colored after repeated fMRI testing sessions”^3^).

While this study lacked power for the group comparison, Hubbard et al. (2005a) took advantage of individual differences between synesthetes. Indeed, the reports of the associations between letters and colors differ between synesthetes, possibly corresponding to different strengths of associations. We may expect that color areas are more activated in synesthetes with stronger associations. Hubbard et al. (2005a) reported a significant correlation between the differential response to graphemes in V4 and the strength of synesthetic association measured with a crowding task. However, this correlation was based on only six data points; they reported a correlation *r*-value = 0.66 with *p* < 0.05, while using the data from their Figure 6 we computed *r* = 0.77 with *p* = 0.075 (Pearson correlation; *p* = 0.05 for Spearman rank correlation); again the correlation is anyway not robust to any cross-validation procedure (like removing JAC). Moreover, the crowding task is probably not a correct measure of synesthetic strength. Ramachandran and Hubbard (2001) had proposed that synesthetes had a higher performance because the synesthetic color could help synesthetes guessing which grapheme was presented (hypothesis of a pre-crowding link between the shape of the letter and synesthetic color). However, superior performance when present seems, in fact, related to the ability to actually identify the letter (failure of crowding on some trials: Ward et al., 2007), not to the strength or quality of synesthetic associations. Hubbard et al. (2005a) also measured the performance of synesthetes in a visual search task, which they had promoted as an objective measure of the synesthetic experience. Surprisingly, they did not report the correlation value between the scores measured for this task and the BOLD response in V4 ([6, Selective reporting]). We computed this correlation using the data points of their Figure 3, and found *r* = 0.12 (*p* = 0.82). Such a result should have led the authors to reject their hypothesis on the role of V4 in synesthesia.

Our conclusion: this study did not demonstrate any correlation between brain signals and individual differences *related to synesthetic behavior*, and did not prove any significant difference between synesthetes and controls (unless basing the conclusion on a one-tailed test, that is making the assumption of a larger activation in synesthetes: [4, a hypothesis is not an *a priori*]).

***Rich 2006: seven synesthetes and seven controls, voxelwise statistics in ROI.*** Rich et al. (2006) presented letters visually to elicit synesthetic colors in seven grapheme–color synesthetes ([1, Sample size]). Control stimuli were squares the same size and gray level as the letters (block design). Using fMRI (3T), they performed analyses only within an ROI defined as the region of the ventral occipital cortex that responded more to colored than grayscale Mondrians, identified at the group level (all subjects; spatially smooth data in a standard SPM normalized brain) using a weak statistic threshold (uncorrected *p* = 0.05), with the idea that this ROI contained rather than defined color specific regions. They corrected for the number of comparisons made in these ROIs, not the number of voxels in the whole brain (“small volume correction” procedure; peak height of probably *t*-statistics, 8 mm FWHM smoothing). Within the ROI, they observed a stronger BOLD signal in the left medial lingual gyrus for letters than squares (*p* = 0.008; Rich et al. (2006) supposed that this activation was not in V4, since it was about 2 cm medial to typical V4 coordinates). They did not observe any significant voxel when they performed the same comparison in a group of seven controls ([2, Accepting the Null]) but they did not compute any between-group statistics. They also tested color imagination the same way and disclosed a different group of activated voxels on the right side in both six synesthetes and the seven controls. They did not compare controls to synesthetes and did not compare the synesthetic and imagination conditions.

Our conclusion: if we make the assumption that the experience of synesthetic colors must involve part of the ventral occipital cortex (involved in color processing), in that case it is more likely to involve the left medial lingual gyrus than V4. The authors reached a similar conclusion except for the conditional reasoning ([4, a hypothesis is not an *a priori*]). The results further suggest that if the activation in the left medial lingual gyrus is related to the experience of colored synesthesia, it does not result from imagining colors, but the direct comparison between both conditions is missing to reach such a conclusion.

***Rouw 2007: 18 synesthetes and 18 controls, cluster extent statistics.*** Rouw and Scholte (2007) measured with an event-related design the BOLD responses (3T) to graphemes that elicited strong, weak or no synesthetic colors in a group of 18 synesthetes, which they compared to those measured in a group of 18 controls. They computed between-group statistics on cluster extent (pFWE = 0.05; *z*-statistics images were thresholded at *z* = 2.3 corresponding to *p* < 0.01, 11.8 mm FWHM spatial smoothing). For the contrast between graphemes that elicited strong or weak synesthetic colors and graphemes that did not, they measured greater activation for synesthetes in four regions (and no region with greater activation in controls): the left frontal cortex, right cerebellum, an inferior region in the right middle temporal gyrus posteriorly located in the right temporal region, and in the (right) fusiform gyrus. This last region was located about 1.5 cm lateral to typical V4 coordinates (no retinotopic mapping and no color localizer were performed). Cluster sizes were about 1 cm^3^ except the frontal cluster (2.9 cm^3^). In none of these regions did the BOLD signal correlate with individual differences of synesthesia, as measured with a questionnaire-based projector/associator score.

Our conclusion is the same as made by Rouw and Scholte (2007): four regions of the brain are potentially involved in the coding of synesthetic colors, including one in the ventral visual cortex (fusiform gyrus). These results should, however, be treated with caution because Rouw and Scholte (2007) used a cluster-forming threshold (*p* < 0.01, unfortunate FSL default value) at which the stationarity hypothesis necessary for the random field theory is not guaranteed (false positive rate is not well controlled; see Hupé, 2015: third section “Pitfalls of MRI statistics: Cluster extent statistics”). There was also no correlation with synesthetic strength as estimated in different subjects. Then, Rouw and Scholte (2007) did not document whether in any of the significant regions, responses were weaker for graphemes that elicited weak synesthetic experiences compared to those eliciting strong synesthetic experiences. They also did not compute or report the results for the opposite contrast (testing if there was any stronger activation for non-synesthetic graphemes).

***Rouw 2010: 42 synesthetes and 19 controls, cluster extent statistics.*** The same authors went on testing more synesthetes on this paradigm and with the same analysis pipeline, for a total of 42 synesthetes vs. 19 controls (Rouw and Scholte, 2010). This time the comparison between synesthetes and controls revealed three large clusters (between 3.3 and 4.7 cm^3^) of increased BOLD signal in synesthetes (none for controls) for the contrast: one cluster around the intraparietal sulcus (see **Table 2**), extending posterior to the parieto-occipital transition zone and occipital gyrus, and extending anterior to the superior parietal lobe (SPL) and angular gyrus; a second cluster located in the medial part of inferior frontal gyrus and precentral gyrus; a third cluster around the parieto-occipital sulcus, mostly in left precuneus cortex (see **Table 2**).

Our conclusion is the same as Rouw and Scholte (with the same reservations as in their 2007 study): three regions of the brain are potentially involved in the coding of synesthetic colors, none of them in the visual cortex^4^.

Rouw and Scholte did not comment on the different results between the two studies neither in the 2010 study or in their 2011 review, where they treated both results as independent (Rouw et al., 2011). The coordinates of the peak activations in both studies were far apart (>2 cm), and no visualization was provided of the extent of the activations, so it is not possible to conclude whether there was any overlap at all. We do not know how to interpret the discrepancy between both studies. On one hand, the second study was more powerful and included all the data of the first study, so we could be tempted to retain only these results. On the other hand most synesthetes had no matched controls in the 2010 study, so part of the results could be due to different low-level visual statistics of graphemes.

***van Leeuwen 2010: 19 synesthetes and 19 controls, cluster extent statistics.*** van Leeuwen et al. (2010) compared 19 synesthetes and controls using fMRI (3T) in two experiments. In the first experiment they measured the BOLD responses to black graphemes that induced synesthetic colors and responses to graphemes that did not, as well as to false fonts, presented in a block design. They only reported the results obtained for the contrast between inducing and non-inducing graphemes. When correcting for multiple comparisons at the whole brain level (SPM5, cluster-extent statistics, pFWE < 0.05, using a voxel threshold at *p* < 0.001 and a minimal extent of 20 voxels; 10 mm FWHM smoothing), no region was significantly more activated by inducing graphemes specifically in synesthetes (interaction between group and condition). They, however, reported such a trend (*p* = 0.052) for a small cluster of 21 voxels in the right fusiform gyrus, but they had to apply a small volume correction (pSVC) in what they defined as a ventro-occipital color ROI. Their subgroups of synesthetes (according to the “associator/projector” questionnaire) did not differ significantly in the ventro-occipital ROI for the contrast between synesthetic and non-synesthetic graphemes. They also contrasted inducing black graphemes to non-inducing colored graphemes and observed a (corrected) significant difference between synesthetes and controls in the left superior parietal lobule (**Table 2**).

In the second experiment they tested more directly the relationship between real and synesthetic colors with an fMRI adaptation protocol, by presenting successively a black symbol and a colored square. The symbol was either a letter inducing a synesthetic color, the same as, or different than the color of the square, or a symbol that did not elicit any synesthetic color. If synesthetic and real colors share some common neural representation, adaptation of the BOLD signal should occur only when the synesthetic and real colors are congruent, and for synesthetes only. The magnitude of adaptation effects are usually small, and to be observed they require that the sequence of event types and stimuli be carefully controlled and counterbalanced (Kourtzi and Kanwisher, 2000). This did not seem to be the case in this study, with, for example, the incongruent condition happening twice as often as the congruent one, and with different proportions of prime types and colors in different runs. Such slight imbalance may explain why results were mostly non-consistent, with a larger effect of prime type in controls (for whom prime type was irrelevant) than synesthetes. In any case, the whole brain analysis did not reveal any interaction effect between subjects and synesthetes for the critical comparison of congruent and incongruent pairs, and no such interaction was observed in regions of interest supposed to be preferentially involved in color processing. The comparison within the group of synesthetes revealed significant suppression effects in the right superior frontal gyrus and the right temporal gyrus (including the hippocampus) but no effect in color regions. When contrasting synesthetic to non-synesthetic stimuli as in their experiment I there was no significant difference between synesthetes and controls in the whole brain analysis as well as in the color ROIs.

Our conclusion: no statistical difference between synesthetes and controls for the contrast specific to synesthetic colors. van Leeuwen et al. (2010) wrote that their results “confirm the role of ventral-occipital color areas in synesthetic color experience,” while, in fact, their result is only *compatible* with such hypothesis ([4, a hypothesis is not an *a priori*]), and only in their Experiment I, not their Experiment II. Synesthetes had a stronger activation than controls in the parietal cortex, for which we do not have any interpretation because it was obtained for the contrast between synesthetic and real colors. van Leeuwen et al. (2010) proposed that it was due to binding and was compatible with the results of Weiss et al. (2005). But in that case this should have been observed also for the contrast against non-inducing graphemes (that was not the case) and against false fonts (that was not reported), as reported by Weiss et al. (2005) in this region for the contrast between synesthetic and non-synesthetic letters. van Leeuwen et al. (2010) also concluded that synesthesia induced suppression effects in the right temporal and frontal cortex even though the interaction with the control group was no significant ([2, Accepting the Null]). This study did not show any evidence of shared neural correlates between synesthetic and real colors (as concluded by van Leeuwen et al., 2010). The conclusions were similar when taking individual differences into account (associators, projector, and mental screen projectors, based on the responses to a questionnaire).

***Hupé 2012: 10 synesthetes, ROI analysis.*** The authors, Hupé et al. (2012b) measured the BOLD responses (3T) of graphemes inducing synesthetic colors, compared to non-synesthetic false-fonts stimuli (event-related design) in visual areas defined with fMRI retinotopic mapping as well as in individual ROIs of maximum color sensitivity (Mondrian protocol). Retinotopic areas V1, V2, V3, and V4 (but not V3a) were on average more activated across the 10 synesthetes by colored Mondrians and by letters. BOLD signal (beta weights) was significantly larger (*p* < 0.05) for letters than for false fonts on the left side in ventral V1–V4, as well as on both sides and also dorsally in V1 and V2. This signal was, however, weak and not larger than measured for the fixation point, except in left V4. Modulation by letters and false fonts was, on average, almost absent in colored areas of maximal response to colored Mondrians (many of these ROIs were not in retinotopic V4). Importantly, they could not observe any positive or significant correlation between signals (possibly related to synesthetic colors) in these color ROIs as well as retinotopic areas and the strength of synesthetic associations measured in each subject with a psychophysics task (synesthetic Stroop task). If anything, the correlation was negative, so the result could not be attributed to a lack of power (in comparison to other published studies). This suggested that the small and distributed differences between letters and false fonts were not related to synesthesia.

The authors further tested the possibility of shared representation of real and synesthetic colors with an adaptation protocol quite similar to that of van Leeuwen et al. (2010), in 9 of their 10 synesthetes (Hupé et al., 2012c). They designed their selection of synesthetes as well as their protocol so each synesthete had for each of four colors two letters inducing about the same synesthetic color. They mixed black graphemes inducing colors with colored false font stimuli in order to create pairs of stimuli made of either two letters, two false fonts or one of each. They did not observe any significant adaptation for synesthetic colors in retinotopic V4 or color ROIs. However, they also did not observe any systematic color adaptation in V4 or in color ROIs, so they could not test rigorously the hypothesis of adaptation across real and synesthetic colors.

***Sinke 2012: 18 synesthetes and 18 controls, two-tailed voxelwise peak statistics.*** Sinke et al. (2012) compared the BOLD responses (1.5T) to graphemes and false-fonts (event-related design) of 18 grapheme–color synesthetes and 18 controls (matched, in particular, for mental imagery as estimated with the “Vividness of Visual Imagery Questionnaire” – VVIQ). Both types of stimuli were presented in an event-related protocol, either in black or in color (same colors used for all subjects, not matched to the synesthete’s colors). The group analysis revealed no interaction between stimulus and group (SPM5, peak heights of *F*-values at pFWE < 0.05, additional criterion of 20 voxels minimal extent; 12 mm FWHM smoothing). They observed for letters plus false fonts versus implicit baseline (fixation cross) a main effect of group, with differences of BOLD responses in the left inferior parietal lobule (IPL; the direction of the effect was not indicated).

Our conclusion (same as made by Sinke et al., 2012): no statistical difference between synesthetes and controls for the contrast specific to synesthetic colors. Activation was different in synesthetes and controls in the left parietal cortex (see **Table 2**). We do not have any interpretation for this effect because it was obtained for the common activation by letters and false fonts. Sinke et al. (2012) proposed that the absence of difference in V4 between synesthetes and controls could be due to their matching of VVIQ scores in both groups. However, evidence of any correlation between VVIQ scores and V4 activity does not exist, and whether VVIQ scores did differ between groups in the other studies was not tested.

***O’Hanlon 2013: 13 synesthetes and 11 controls, cluster extent statistics.*** O’Hanlon et al. (2013) compared the BOLD responses (3T) to graphemes and false-fonts in 13 synesthetes and 11 controls in a block design. The group analysis revealed significant interactions between stimulus and group in 14 regions (Afni^5^ software, cluster-extent statistics, pFWE = 0.01 computed with Monte-Carlo simulation, images were thresholded at *p* < 0.0005^6^; significant clusters had a minimal extent of 134 mm^3^ or voxels; 7 mm FWHM smoothing). Interactions were, however, not due in any area to a stronger response to graphemes in synesthetes but not controls. In three regions the average BOLD response to graphemes was even negative and lower in synesthetes than controls (in the left and right IPLs and the left transverse temporal gyrus). But in four other clusters the significant difference between synesthetes and controls was obtained for the response to false-fonts.

O’Hanlon et al. (2013) also measured the response in nine regions of interest defined based on possible GM differences between controls and synesthetes (VBM Analysis, see Appendix: Structural Studies). They computed in each ROI the interaction between stimulus and group, and since they made nine comparisons they set their significance threshold to 0.0056 (Bonferroni correction, 0.05/9). None of the interactions reached that threshold, even close (minimum *p*-value was 0.018).

Our conclusion: the whole brain analysis showed no specific activation by synesthetic colors in synesthetes. The pattern of responses in controls and synesthetes was, however, different in many brain regions, for either graphemes or false fonts, with no apparent logic. O’Hanlon et al. (2013) insisted on the significant decrease of the BOLD signal for letters in synesthetes observed in three regions (none in the visual cortex) but they did not propose any explanation for why decreasing should be observed in these particular regions. The analysis constrained on possible structural differences between synesthetes and controls did not reveal any functional difference [O’Hanlon et al. (2013) concluded that there was also a significant decrease of the BOLD signal for letters, but “significance” was based on what we identified as a logical error – see our comment on the Frontiers website].

***Tomson 2013: 16 synesthetes and 15 controls, voxelwise FDR statistics.*** Tomson et al. (2013) compared the BOLD responses (3T) of 16 “colored sequence” synesthetes (who may associate colors to letters, numbers, weekdays, and months but not to tones) and 15 controls to 6^∘^ tall graphemes and pseudographemes (created by manipulating graphemes in Photoshop; in the examples shown pseudographemes had many more edges than letters), in a block design. They only reported the between group comparisons when contrasting either graphemes or pseudographemes against rest, and observed no significant difference (SPM8, correction for multiple comparisons at FDR = 0.05, 8 mm FWHM smoothing). They did not report the critical contrast for synesthesia between graphemes and pseudographemes but looked at the thresholds maps obtained in both groups independently when contrasting pseudographemes against graphemes ([2, Accepting the Null]). They insisted on the absence of any activity in synesthetes for these stimuli in parietal regions.

Our conclusion: no statistical difference between synesthetes and controls for the contrast specific to synesthetic colors. No other differences between synesthetes and controls.

***Melero 2014: 10 synesthetes and 10 controls.*** We apologize for not including the study by Melero et al. (2014) published after we had written our review. We do not think that their results contradict the main message of this review.

##### Single-case studies

***Aleman 2001: one synesthete, auditory stimuli.*** Aleman et al. (2001) studied with 1.5T fMRI a single synesthete, for whom hearing and producing a word resulted in seeing the word in her mind’s eye with a particular color. Measuring the contrast between heard or produced words and pure tones (voxelwise statistics, no indication of spatial smoothing), the authors reported a few significant voxels scattered in anatomically defined V1, but only when correcting for the number of voxels in V1 (Bonferroni correction). These voxels were not significant anymore when correcting (Bonferroni) for the number of voxels in the whole brain. At that over-conservative threshold a few significant voxels remained scattered in the brain, and the authors indicate that some of them were located in the posterior–inferior temporal (PIT) cortex, thus possibly involving color processing. The figures provided by the authors are not convincing that the few significant voxels “peppered in the brain” (as already observed by Blake et al., 2005) are something else but noise.

***Weiss 2001: one synesthete, visual stimuli.*** Weiss et al. (2001) studied a synesthete who experienced color for names of personally familiar people. When contrasting the BOLD signal (1.5T fMRI) for blocks of familiar against unfamiliar names presented visually, they observed a significant increase in the retrosplenial cortex and the extra-striate cortex bilaterally (SPM, cluster-extent statistics pFWE < 0.05, using an initial threshold at *p* < 0.001). All names were presented either in gray or in random color. When contrasting colored against gray stimuli, the fusiform gyrus was activated bilaterally. There was no overlap with the extra-striate region activated by familiar names (more dorsal and lateral).

Our conclusion: the neural mechanisms of synesthetic colors “differ from those associated with color perception,” as proposed by Weiss et al. (2001).

***Elias 2003: one synesthete and one matched control, auditory and visual stimuli.*** Elias et al. (2003) studied a grapheme–color synesthete and a “semantic” control (a cross-stitcher) with 1.5 T fMRI in different visual and auditory tasks. The analysis was non-standard, not explained enough (we do not know what conditions if any were contrasted) and included subjective criteria (like false activations eliminated by visually inspecting the signal time course). Elias et al. (2003) reported that the synesthete and the semantic control exhibited similar activations in a color–number Stroop task, for both the congruent and incongruent conditions, but that patterns were different for the visual and auditory arithmetic tasks; no direct comparison was done ([2, Accepting the Null]).

***Sperling 2006: four synesthetes, visual stimuli.*** Sperling et al. (2006) compared the BOLD responses (1.5 T) of four grapheme–color synesthetes to graphemes and colored stimuli. They presented in a block design either three letters (for each synesthete) that evoked color experiences or three letters that evoked only gray/transparent experiences (all letters had therefore some synesthetic quality). For one subject they showed the activations thresholded at *p* = 0.05 (FDR correction for multiple comparison) on a flat cortex reconstruction, obtained for real and synesthetic colors, contrasted against the baseline. The activations were widespread within retinotopic areas, especially for the colored Mondrians, as well as in the frontal cortex, as expected given the lack of specificity of the contrast. Their point was to show that a small portion of retinotopic V4 (defined with fMRI retinotopic mapping) was significantly activated for both stimuli with either real or synesthetic colors. Specificity of such overlap would, however, require that different results be obtained for letters inducing no synesthetic color. Corresponding flat maps were not provided. The authors reported in their Methods section that they defined these overlapping significant clusters for colors and letters against rest within retinotopic V4 as the ROI in each subject, and then contrasted the average response for colored (and synesthetic) against achromatic (and non-inducing) stimuli. They indicated that for two subjects these contrasts were significant (*p* < 0.05). Unfortunately, we could not evaluate the strength of this result because the published tables did not seem to correspond to this result. In their Table 2, Sperling et al. (2006) provided for these two subjects the Talairach coordinates of the center of mass of the activations obtained within V4 independently (centers of mass were 3–8 mm apart) in the color mapping and the synesthesia experiments (real or synesthetic color against baseline). The responses to non-synesthetic letters did not seem to be significantly weaker than the responses to synesthetic letters in the synesthetic V4 ROI^7^. The values reported in their Table 2 for the “V4 ROI” were the same as those in their Table 1 for their “V4/V8” ROI. Sperling et al. (2006) also performed a whole brain analysis in each subject for the contrast of synesthetic colors. They observed significant clusters of voxels in three of their four subjects, mostly within the inferior frontal cortex. There was, however, a lack of overlap between subjects, and these differences were observed at an uncorrected *p*-value = 0.05.

Our conclusion: we could not figure out what the results were exactly. For two subjects Sperling et al. (2006) reported that a few voxels within retinotopic V4 that responded strongly to real colors responded also strongly to letters inducing synesthetic colors but significantly less to non-inducing letters. We inferred from the published tables that a few voxels within V4 that responded strongly to inducing letters responded less to non-inducing letters (average difference of beta values between the two conditions was 1.72, range = [0.4 2.79]), but without any clear evidence either of significance or involvement in real color processing.

***Steven 2006, Niccolai 2012: one blind synesthete, auditory stimuli.*** Steven et al. (2006) and Niccolai et al. (2012a) collected fMRI images on a late-blind subject, JF, who reports having kept his synesthetic visual experiences. JF experiences days of the week and months as colored, rectangular shapes, spatially organized (sequence space synesthesia). Other time words (like “morning” or “Easter”) have also a shape and a spatial position, but not color. In the first study, Steven et al. (2006) had JF listening in a 1.5T scanner either to time words triggering synesthetic colors or other words, whose frequency of usage was matched. JF showed activations when contrasting both conditions in the visual cortex, including Brodmann areas 17 and 18, notably at MNI coordinates corresponding to V4 (FSL, cluster extent statistics, pFWE < 0.01, height threshold *z* > 2.3; spatial smoothing was indicated 3 mm FWHM, but this may have been sigma, which is the parameter to be specified in FSL, corresponding to FWHM = 7 mm). No such activation was observed for both a late-blind and a sighted non-synesthetic control (no direct comparison [2, Accepting the Null]). JF also performed a color imagery task, imagining a very familiar colored object (only one stimulus: a red sweater). Activation (when contrasted against rest) was observed in the visual cortex, ventrally and more anterior than for time–words. Unfortunately, no direct comparison was performed between time–words and color imagery, in order to test if the observed pattern of differences was significant ([2, Accepting the Null]). Also, the color imagery task was not performed (or not reported) for the two control subjects. Steven et al. (2006) argued that the activation at the anatomical coordinates of V4 for time words but not color imagery suggested that the synesthetic experience of color by JF was similar to the perception of color in sighted observers. This strong statement is, however, not grounded on a statistical basis, as explained above. Moreover, the activation observed from V1–V4 could be due to the experience of shape rather than color. In any case, the results did not show any functional overlap for the experience of synesthetic and imagery colors (of course no comparison for real colors could be done for this blind subject).

In the second fMRI (3T) study of JF, Niccolai et al. (2012a) tried to dissociate the BOLD activations due to synesthetic shapes and colors. They used the same time words as before to elicit both synesthetic shapes and colors; they also used time words that elicited only shapes, and time words that elicited no image. When contrasting the color-and-shape time words against non-synesthetic words, activation was again observed in the visual cortex, including around the typical coordinates of V4 (SPM8, FDR *p* < 0.05 – the activation was not significant at their predefined threshold: cluster extent statistics, pFWE < 0.05, height threshold *p* < 0.001; minimum cluster extent = 50; 5 mm FWHM spatial smoothing). Activation was also observed in the visual cortex, but not V4, for shape-only time words. However, the key contrast between color-and-shape and shape-only time words did not reveal any difference over the whole brain, and the V4 activation was not significant anymore when contrasting color-and-shape and shape-only time words together against control words (there were significant activations in the superior occipital gyrus and the intra-parietal cortex). Therefore, the activation in V4 cannot be attributed with confidence to the synesthetic experience of either color or shape (unless [2, Accepting the Null]).

Our conclusion: this rare and thorough single-case study of a blind person experiencing synesthetic colors revealed activations within the visual cortex, suggesting functional reorganization. We do not think that the data could ascertain that the reported activations in V4 corresponded to the synesthetic experience of color.

***Bor 2007: one Asperger synesthete and 14 controls, auditory stimuli.*** Famous synesthete DT (Daniel Tammet) experiences numbers as organized in a 3D mental space, also varying in size, texture, form, and color. Bor et al. (2007) measured the BOLD signal (3T) during a digit span task, with two critical conditions: easy and difficult lists of numbers. One critical result was an absence of difference measured in the brain of DT for these two conditions during the encoding phase (when subjects had to listen and memorize lists of spoken numbers) and an increase for controls in the lateral prefrontal cortex (LPFC) for the difficult series (SPM 99, voxelwise statistics pFDR < 0.05, 20 voxels minimum cluster size). When computing interaction effects, this difference was close to significance in the independently defined ventral LPFC ROI. Such a result fits nicely with the subjective reports and behavioral memory performances of DT for whom memorizing any sequence of numbers is very easy because of their 3D mental organization. Contrasting both conditions against the delay phase, DT had a significantly larger activation in at least the left LPFC, suggesting additional “chunking processes” in DT (Bor et al., 2007). However, no other activation (in the visual or parietal cortex) was observed for DT in comparison to controls either in the whole brain analysis or in “the V4 ROI taken from Nunn et al. (2002) or in anatomically defined visual ROIs (all *p* > 0.1),” where one could expect to observe correlates of DT’s strong mental imagery.

Our conclusion: this thorough single-case study of an individual with reports of particularly strong experience of colored images for numbers did not reveal any correlate of this subjective experience within the visual cortex. The design of the experience was, however, not directly comparable to other studies that contrasted similar stimuli inducing or not a synesthetic experience.

#### fMRI STUDIES, EXPERIMENTS ON OTHER TYPES OF SYNESTHESIA

***Tang 2008: Number form synesthesia, 10 synesthetes and 10 controls, visual stimuli.*** Tang et al. (2008) compared fMRI activations in two groups of 10 “number forms” synesthetes and 10 controls performing in a 3T scanner either a number magnitude or a number order task. These synesthetes report experiencing a specific spatial organization (in their mind’s eye) for sequences of numbers, as well as often for non-quantitative sequences like months or letters, meaning that this synesthesia is related to the ordinal representation of numbers rather than their cardinality (magnitude). Tang et al. (2008) selected synesthetes who experienced a general direction from left-to-right for small numbers (<10). They contrasted two ordinal tasks: subject had to judge the ordinal position of a number within a line of three to five items (1 numeral and 2 to 4 “X”), either starting from the left (therefore in a direction roughly compatible with the direction of their number line) or starting from the right. Subjects also ran a cardinal task with the same stimuli where they had to decide whether the numeral corresponded to the number of items in the display. They reported stronger activations (SPM 5, cluster extent FDR statistics^8^, images thresholded at *p* < 0.001; 8 mm FWHM smoothing) in synesthetes than controls for the L–R (congruent) task compared to the R–L (incongruent) task in many regions all over the brain, including along the banks of the posterior IPS, supposedly involved in spatial processing (Hubbard et al., 2005b). Tang et al. (2008) proposed that the experience of this type of synesthesia induces neural activity in the regions normally involved in the experience of the concurrent (the spatial form), but only in very specific conditions, when synesthetes make ordinal judgments on stimuli spatially congruent with their number line. It is not clear to us why there should be such specificity. First of all, the stimuli were not exactly congruent with number lines (some synesthetes had oblique directions or direction change within the 1–10 sequence). Second, we may have expected stronger activation for conflicting stimuli (like in Stroop tasks) – no brain region showed such an effect. Third, since synesthetic associations are automatic (but maybe not so much for number lines: Price and Mattingley, 2013), we may have expected the posterior IPS to be activated whenever numbers were presented. This was not the case even when considering the group of synesthetes alone and at a lenient threshold (uncorrected *p* < 0.001, no FDR correction: see their Table 2). The statistical comparison with controls for this main effect was not provided.

Our conclusion: even though this study was not designed to identify the neural correlates of the subjective experience of number-line (no control stimulus), the posterior IPS, supposedly involved in spatial processes relevant to number (Hubbard et al., 2005b) was not activated by numbers in synesthetes. The reported difference between synesthetes and controls in the posterior IPS when contrasting conflicting and non-conflicting stimuli is hard to interpret; it lacked specificity (other brain regions with the same effect) and was potentially a false-positive result (no clear indication that the FDR procedure was applied and 8 mm smoothing might not be sufficient for cluster extent statistics to be reliable – see Hupé, 2015: third section “Pitfalls of MRI statistics: False Discovery Rate”).

***Jones 2011: Word–taste synesthesia, two single-case synesthetes and 10 controls, visual stimuli.*** Jones et al. (2011) compared the BOLD signal (1.5T) of two synesthetes experiencing tastes for words and of 10 controls. They contrasted the affective nature of the synesthetic experience, dividing words in four lists (pleasant, unpleasant, neutral, no taste) for one of the synesthetes (JIW). Prior to scanning, five controls were trained to associate these words with faces depicting disgusting, happy, and neutral expressions matching JIW associations. Five controls learned mismatched associations (but the authors did not report whether this had any effect). The other synesthete was presented with the same stimuli. Affective categories were therefore not balanced (different synesthetes make different associations) and direct comparisons could not be done. The main result was the comparison of JIW to the 10 synesthetes’ BOLD responses. The BOLD signal was not significantly greater for “tasty” than “tasteless” words in primary or associative regions encoding taste/flavor information (insula) either in JIW alone or in comparison to controls (SPM8, voxelwise *t*-statistics, pFWE < 0.05, additional extent threshold of 10 voxels; 8 mm FWHM smoothing; also no difference when applying SVC in these regions), contrary to the hypothesis of neural activity triggered by synesthesia in the regions normally involved in the experience of the concurrent [Jones et al. (2011) did not take these results into account in their conclusions]. The only difference between JIW and controls was observed in the precuneus, which was, however, not revealed by the whole brain analysis performed in JIW alone (as expected if this was related to the synesthetic experience). Contrasting emotional to neutral tasting words revealed no significant cluster in the whole brain analysis either in JIW alone or in comparison to controls. Jones et al. (2011) reported activation in the left anterior insula in JIW alone that reached significance when applying small volume correction [leading Jones et al. (2011) to conclude that word–taste synesthesia recruited regions involved in taste and emotion processing (4, a hypothesis is not an *a priori*)]. This activation was larger (SVC) than in controls only for the specific contrast of unpleasant against neutral words, even though JIW had no significant difference for pleasant and unpleasant words. The critical comparison with controls who had learned the specific associations was not reported ([6, selective reporting]). Since each synesthete had evaluated the intensity of the synesthetic taste for each word, Jones et al. (2011) also reported the correlation between intensity and BOLD signal. There was no significant cluster in the insula as well as no overlap of significant clusters between both subjects (both had a significant cluster in the precuneus, but about 3 cm apart and on opposite sides) as well as no overlap for JIW with the precuneus cluster (about 2.5 cm away) revealed by the comparison with control subjects.

Our conclusion: no evidence of neural correlates of the subjective experience of synesthetic taste, notably in the regions involved in taste processing.

#### EEG, MEG

***Baron-Cohen 1987: EEG, single-case grapheme–color synesthete, auditory stimuli.*** The first EEG recording of a grapheme–color synesthete hearing words was obtained by Baron-Cohen et al. (1987), who did not observe any abnormality of signals.

***Schiltz 1999: EEG, 17 grapheme–color synesthetes and 17 controls, visual stimuli.*** Schiltz et al. (1999) recorded the EEG signals of 17 grapheme–color synesthetes and as many control subjects. They measured the even-related potentials (ERP) triggered by six letters and four numbers presented visually for 300 ms. They observed large between-groups differences that were significant only over frontal and parietal electrodes (29 electrodes system) and within the 200–300 ms average time-window. It is difficult to infer what type of processing was different between both groups, since no difference was observed over occipital electrodes, and, critically, they did not record and compare ERPs for visual stimuli inducing no synesthetic color.

***Beeli 2008: EEG, 13 grapheme–color synesthetes and 13 controls, auditory stimuli.*** Beeli et al. (2008) recorded over 30 channels the EEG signals of 13 right-hand “colored-hearing” (in fact, probably all grapheme–color) synesthetes and controls. Subjects listened to 300 stimuli (words, pseudowords, and letters), all inducing the experience of color in synesthetes, with eyes closed. Amplitudes and latencies of the P1, N1, and P2 ERP components were taken from the Cz electrode and compared between groups. ERPs were comparable in both groups, not reproducing the large differences observed by Schiltz et al. (1999) with visual stimuli, with yet slightly longer (10 ms on average) latencies of the N1 and P2 peaks in synesthetes, as well as a smaller amplitude of P2. LORETA source reconstruction around the N1 and P2 peaks revealed significantly (Bonferroni corrected) larger signals in synesthetes at a few estimated sources. For N1, the estimated signal was larger in the left PIT for letters but not for words and pseudowords (combined; there was yet a tendency: *p* < 0.1) and in the ventromedial orbitofrontal cortex (also for letters only). These two sources were significant at P2 for words, while letters this time evoked larger signals in synesthetes in the left superior frontal gyrus, the left, and the right intraparietal sulcus. This study was suggestive that the processing of synesthetic colors could start as early as 122 ms in color regions (PIT). However, the results were not fully consistent for letters and words and several differences between groups may be difficult to account for, like latency shifts and differences in the orbitofrontal cortex. Moreover, it is not clear how these effects may interact. If the observed latency shift corresponds to a true latency difference, then source reconstructions at a given time point are expected to be different (but the opposite reasoning could be done). The major limitation of this study was the absence of control stimuli, like in the study by Schiltz et al. (1999).

***Goller 2008: EEG, 10 colored hearing synesthetes and 10 controls, auditory stimuli.*** Goller et al. (2008) recorded ERPs (31 electrodes system) of 10 colored-hearing synesthetes and 10 controls listening to five pure tones. They observed smaller amplitude of the N1 component with no interaction with electrode site. Closer inspection of occipital electrodes revealed no evidence that tones evoked a visual potential in synesthetes. Further analysis of two of these synesthetes experiencing also auditory experience for colors, as well at the comparison with two former single-case studies (Rizzo and Eslinger, 1989; Rao et al., 2007), did not reveal any consistent pattern of results. Like in previous EEG studies, there was no comparison with non-synesthetic stimuli, and power was low (especially because sex and right-handedness was not matched between both groups).

***Brang 2010: MEG, four grapheme–color synesthetes and four controls, visual stimuli.*** Brang et al. (2010) used MEG to measure the responses to letters and numbers in four grapheme–color synesthetes ([1-Sample size]). However, they did not report the comparison for the responses to false font stimuli, which they had included in their protocol ([6, selective reporting]). They only compared the average response of the four synesthetes and controls in “V4” and “grapheme area” regions of interest, defined with MEG (source reconstruction) by applying strong *a priori* ([4, a hypothesis is not an *a priori*]). Performing parametric *t*-tests to compare two “groups” of four subjects is well below any accepted statistical standard. Such a study remains therefore to be done on a large group of subjects, comparing not only synesthetes with controls for the same stimuli, but also for non-inducing stimuli as similar as possible as graphemes, and with a minimum of hypotheses concerning the localization of effects.

***Jäncke 2012: EEG, 11 colored hearing synesthetes and 11 controls, auditory stimuli.*** Jäncke et al. (2012) recorded the EEG signals of 11 colored-hearing synesthetes and 11 controls during a passive MMN (mismatch negativity) task. Subjects were instructed to watch a silent movie while ignoring tones. The standard tone was a piano tone A (440 Hz), presented 60% of the time. Deviants were either close to the standard (438 Hz: slightly mistuned A; 422 Hz: mistuned G#; 416 Hz: G#, one semitone deviant) in order to elicit similar colors for synesthetes or further away (264 Hz: piano tone C, nine semitone deviant). Each deviant occurred 10%. Significant MMNs at around 150 ms were recorded for all deviants and both groups. The amplitude of the MMN was, however, larger in synesthetes for the two largest deviant tone, [one semitone, *t*(20) = 3.9, *p* < 0.001; 9 semitone, *t*(20) = 2.726, *p* < 0.01] suggesting that the larger deviance was due to the synesthetic color being processed preattentively. LORETA source reconstruction suggested the possible involvement of visual areas in synesthetes. The authors were, however, aware that their 32 electrodes system did not allow them to draw firm conclusions concerning intracerebral source localization. One limitation of this study, besides relatively weak power, is the absence of measure of the MMN for control stimuli with no synesthetic quality, so we cannot rule out the possibility that this particular group of synesthetes just had a stronger MMN for stronger deviants, irrespective of synesthesia. Moreover, tone deviance did not match the differences between synesthetic colors. While synesthetes “reported clear, distinct color sensations” while hearing tones A and C, a statistically more reliable difference was obtained for tone G#, but not the mistuned G#, even though five of the 11 synesthetes perceived identical colors for these two tones. Moreover, inspection of their Supplementary Table revealed that the approximate number of colors different than the standard (depending of course on the exact rendering of RGB values) for the four deviants were, respectively, 2, 10, 9, and 11 (with larger distances in color space specifically for the nine semitone difference). This protocol seems, however, promising to detect (if any) early correlates of synesthetic colors, if able to carefully choose synesthetes and tones in order to fully dissociate tone deviance from synesthetic color deviance.

#### OTHER STUDIES

For the comprehensiveness of this review we cite other studies focused on interference effects due to synesthesia (whether the synesthetic experience is congruent or not with the context) rather than the neural correlates of the synesthetic experience, using EEG (Cohen Kadosh et al., 2007; Brang et al., 2008; Gebuis et al., 2009; Teuscher et al., 2010; Brang et al., 2011; Niccolai et al., 2012b), fMRI (Cohen Kadosh et al., 2007; Laeng et al., 2011), Transcranial Magnetic Stimulation (TMS; Esterman et al., 2006; Muggleton et al., 2007) or even Transcranial Direct Current Stimulation (TDCS; Terhune et al., 2011). A few other studies explored the neural mechanisms related to other aspects of synesthesia, like bidirectionality, with TMS (Rothen et al., 2010), or compared synesthetes and controls for EEG signals to non-synesthetic stimuli (Barnett et al., 2008) or for the susceptibility to report phosphenes with TMS stimulation (Terhune et al., 2011).

The description of these studies is beyond the scope of the present review, in particular because their interpretation may be difficult as long as the neural correlates of the synesthetic experience are not known. However, we should say a few words about two influential TMS studies. These studies measured the strength of the interference in a Stroop task with or without TMS over, respectively, the right posterior parietal lobe of two synesthetes (Esterman et al., 2006), and parietal and parieto-occipital regions of five synesthetes (Muggleton et al., 2007). It was reduced^9^ for the seven synesthetes but not abolished. No phenomenological report was requested, so we do not know whether the synesthetic experience of colors was disrupted. Moreover, no other interference task (not involving synesthesia) was performed to test the specificity of the TMS stimulation regarding the synesthetic experience. Terhune et al. (2011) used a similar paradigm with TDCS. The significant effects on synesthetic Stroop interference of TDCS, tested this time over the primary visual cortex, were based on group analyses of five synesthetes only ([1 – Sample size]), performed with parametric tests on biased summary measures, like in the TMS studies (see footnote 9).

### STRUCTURAL STUDIES

#### STRUCTURAL MORPHOMETRY STUDIES

##### Group studies

***Rouw 2007: 18 grapheme–color synesthetes and 18 controls, DTI, uncorrected peak statistics with cluster extent threshold.*** Rouw and Scholte (2007) used DTI (32 directions, 3T) to measure fractional anisotropy (FA) with FSL tools. FA was measured in each voxel. Large values correspond to coherent white matter tracts. Voxelwise statistics were computed only along the tracts of white matter (Tract-Based Spatial Statistics, TBSS). These tracts are based on the mean FA image across subjects thinned to create a “skeleton” (this procedure was designed to overcome “the arbitrariness of the choice of spatial smoothing extent”^10^). They “considered activation significant at a t-value higher than 3, with a minimum cluster size of 40 mm^3^” (corresponding to about six acquired voxels; they indicated that they used permutation tests to evaluate the significance of *t*-values, but did not report any significance value. Note that for a Student test with d.o.f. = 34, *t* = 3 corresponds to one-tailed *p* = 0.0025). They did not report the risk of false positives controlled by this arbitrary threshold (the random field theory cannot be applied to skeletons, which are not random fields. Permutations could be performed to evaluate the minimum cluster size obtained by chance. The authors did not indicate whether this procedure was applied and in this case, at which FWE level. In any case, the only conclusion based on such a test would be that the two groups are not exchangeable, without any possible inference on the mean FA; see Hupé, 2015: third section “Pitfalls of MRI Statistics,” paragraph on Permutation tests). They found that, compared to controls (*n* = 18), grapheme–color synesthetes (*n* = 18) showed greater anisotropic diffusion, meaning more coherent white matter, in the right inferior temporal cortex, in the left superior parietal cortex and a bilateral cluster of higher FA in the superior frontal cortex beneath the central sulcus. They did not find any greater FA in controls. The higher FA in the right inferior temporal cortex (max *t* = 4.8) was particularly interesting because of the possible involvement of this region in color and linguistic processing. Rouw and Scholte (2007) explored several possible correlations with other, independent measures. (1) They observed in this region a correlation with the scores to a projector/associator questionnaire (*p* < 0.009, one-tailed non-parametric Spearman correlation test, assuming a positive correlation [4, a hypothesis is not an *a priori*]; they did not use robust correlations and did not provide bootstrap confidence intervals; Rousselet and Pernet, 2012; no correction was applied for multiple comparisons while four correlations, positive and negative, were computed). (2) They measured the main direction of the white matter tracts in the right inferior temporal cortex, in order to see if higher anisotropy in this region for synesthetes could indicate a different pattern of connections. They detected no difference between synesthetes and controls. (3) An fMRI experiment on the same subjects had revealed a cluster of increased BOLD signal for stimuli that induced strong or weak synesthetic colors, compared to non-inducing stimuli (see Appendix: Functional Studies) also in the right inferior temporal cortex. However, this activation was not in the same gyrus as the increase of FA (and at least 1 cm away). (4) The authors explored the relationship across synesthetes between FA and BOLD signal in these two different regions of the inferior temporal cortex. They measured a significant correlation between FA and the BOLD response to either stimuli that induced strong or weak synesthetic colors (*p* = 0.044 and *p* = 0.023, respectively, one-tailed test implicitly considering a positive correlation [4, a hypothesis is not an *a priori*]), but also a comparable trend between FA and the response to false fonts (*p* = 0.076). These weak correlations were therefore not related to the synesthetic experience (unless [2, Accepting the Null], which the authors did not do but engaged the reader to do).

Our conclusion: nothing can be concluded about the statistical significance of the reported results. As an exploratory analysis, the higher FA value observed in the right inferior temporal cortex of synesthetes is interesting, whether significant or not. Rouw and Scholte (2007) explored in detail whether independent measures could substantiate this observation. This was not the case for the direction of fibers as well as for the BOLD signal related to synesthesia. The only support came from the correlation with the scores of the projector/associator questionnaire. However the result was statistically weak (*p* > 0.05 if using a hypothesis-free two-tailed Spearman test and considering the family of four tests performed) and not robust to cross validation (removing any one of the three values with the highest scores to the questionnaire made the correlation not significant anymore: uncorrected *p* > 0.05, two-tailed Spearman). Finally, the authors indicated that they removed the answers to one of the questions of the questionnaire because of lack of consistency with the other answers; we do not know whether any correlation is still present when including all answers. Lack of consistency may reflect the difficulty to capture subjective differences with the questionnaire (in our experience, we had many subjects describing seeing the colors both on the letter written on the page, suggesting a projector-like experience, as well as in their mind’s eye, suggesting associator-like experience. Both reports may appear contradictory only to a non-synesthete asking the questions).

***Jäncke 2009: 24 grapheme–color synesthetes and 24 controls, GM analysis and DTI (28 subjects), one-tailed peak statistics.*** Jäncke et al. (2009) acquired T1-weighted structural images at 3T to compare 24 grapheme–color synesthetes with 24 controls. They used DTI (15 directions) in a subsample of 28 subjects.

For DTI, they “adhered as closely as possible to [the] analysis” by Rouw and Scholte (2007) to try to replicate their results. They only looked for higher values in synesthetes (voxelwise *t*-test). They found no difference when correcting for multiple comparisons (pFWE < 0.05; they did not indicate the correction method). When using an arbitrary, lenient threshold (*p* < 0.01, minimal extent 30 voxels or mm^3^), they identified four clusters, none in the right inferior temporal cortex, therefore not replicating the results of Rouw and Scholte (2007). The largest difference (voxel with *t* = 5.55) was observed on the left side in the splenium of the corpus callosum.

They measured the volume, thickness, and surface of the cortical ribbon as reconstructed with FreeSurfer^11^ in 48 subjects. They only looked for higher values in synesthetes, including in their GLM the whole volume, mean thickness, or whole surface, respectively, as cofactors. They found no significant difference (pFWE < 0.05; 13 mm FWHM smoothing). When using an arbitrary, lenient threshold (*p* < 0.01, minimal extent 50 vertices), they reported increased values in the fusiform gyrus which they supposed could be close to V4α (Bartels and Zeki, 2000); the peak difference in the right fusiform gyrus in this study (*t* = 3.52, maximum *t*-value measured at a vertex) was more anteriorly located than that of Weiss and Fink (2009). Altogether, this exploratory analysis revealed 23 clusters. The largest difference measured at a vertex (*t* = 3.83) was observed for cortical thickness in the right calcarine sulcus (V2)/lingual gyrus.

Our conclusion: no statistically significant structural differences between synesthetes and controls. They reported all weak differences observed between both groups, to be potentially used in meta-studies. Unfortunately, they only reported the larger values for synesthetes, not for controls.

***Weiss 2009: 18 grapheme–color synesthetes and 18 controls, VBM-GM, peak statistics and within ROI statistics.*** Weiss and Fink (2009) used optimized VBM (SPM2) at 3T to compare the local GM volume of 18 grapheme–color synesthetes and 18 controls. The whole brain analysis revealed no significant difference (pFWE < 0.05, 12 mm FWHM smoothing; mean corrected age and global brain volume were used in the GLM as nuisance covariates). WM differences were not tested. Hypothesis-driven analyses in spherical search volumes (size not indicated) revealed a higher volume of local GM in the left (but not the right) caudal intra-parietal sulcus (cIPS, *t* = 3.84, pSVC < 0.05; extent not indicated) and in the right (but not the left) fusiform gyrus (FG, *t* = 2.94, pSVC < 0.05; extent not indicated). They also performed a whole brain analysis (*p* < 0.001, uncorrected, corresponding to *t* > 3.6) that revealed an additional area of GM difference in the left superior temporal sulcus (STS, *t* = 3.91; extent not indicated). Since they reported only *t*-values we suppose that they only tested higher GM volume in synesthetes. They found no significant correlation between local GM volume in the FG and cIPS (they did not test the STS) and the scores to the projector/associator scale. Local volume was, however, correlated between FG and cIPS (*p* < 0.001).

Our conclusion: no statistically significant GM differences between synesthetes and controls. If any, the most likely difference was observed in the left STS and the second most likely in the left caudal IPS.

***Rouw 2010: 42 grapheme–color synesthetes and 42 controls, VBM-GM, uncorrected cluster extent statistics and ROI statistics.*** Rouw and Scholte (2010) used optimized VBM (FSL) at 3T to compare the GM of 42 synesthetes and 42 controls. They reported for their whole brain analysis clusters containing at least 200 contiguous voxels with uncorrected *p* < 0.05 (permutation test; 9.4 mm FWHM smoothing). They did not indicate including brain volume as a cofactor in their analysis; their measure is therefore related to both local and global GM volume. They did not report any FWE statistic, and the cluster-forming threshold was anyway too low to correctly control the rate of false positives. Clusters were identified based on a 3-level ANOVA (42 controls/26 associators/16 projectors; one question of the questionnaire was removed like in their 2007 study). *T*-tests were performed *post hoc* to identify the most likely origin of these differences. The ANOVA revealed six clusters, two of them related to synesthesia independently of the projector/associator classification: they observed greater GM in the left superior parietal cortex of synesthetes (*t* = 3.5 for both associators and projectors against controls) and lower GM in the cingulate sulcus (*t* = 2.7 for projectors and *t* = 3.9 for associators against controls). They did not find any region that differentiated both synesthetes from controls and associators from projectors (based either on categorization or the score to the questionnaire).

Rouw and Scholte (2010) also performed ROI analyses in the anterior intraparietal sulcus (human intraparietal areas 1 and 2, hIP1 and hIP2) and in the fusiform gyrus, divided in four parts along the posterior–anterior axis (occipital, occipito-temporal, posterior and anterior temporal divisions). Among these 12 ROIs, the largest differences between synesthetes and controls were observed in the left hIP2, with larger GM in synesthetes (*t* = 1.97, uncorrected *p* = 0.053; groups had different variances), and in the left temporal fusiform gyrus, with larger GM this time in controls (*t* = 2.03, uncorrected *p* = 0.046). These differences did not survive correction for multiple comparisons.

Rouw and Scholte (2010) also looked for specific differences between projectors and associators. Their statistical model was, however, overparameterized since they used the P/A score as a covariate and the PA classification (based on P/A score) as an independent variable. They also did not indicate any FWE level for their cluster extent statistics. The maximum *t*-value (*t* = 4.5) was measured in a voxel of the cerebellum.

Our conclusion: no statistically significant GM differences were reported between synesthetes and controls, or between projectors and associators. The whole brain analyses were inadequate and did not provide any useful information. ROI analysis, though powerful (84 subjects) revealed only a trend for larger GM in the left IPS of synesthetes; an opposite trend was observed in the left fusiform gyrus.

***Banissy 2012: nine tone and grapheme–color synesthetes (two left handed) and 42 right-handed controls, VBM-GM, peak statistics and within ROI statistics.*** Banissy et al. (2012) compared the GM at 1.5T of nine synesthetes who experienced color for both graphemes and tones with 42 controls. The whole brain analysis revealed no difference (SPM8, voxelwise *t*-tests, pFWE < 0.05; smoothing was not indicated; gender and age but not brain size were used as covariates).

They defined eight spheres (12-mm radius) as regions of interest, in the fusiform gyrus, MT/V5, the middle temporal gyrus and the intraparietal sulcus. They used the coordinates of Weiss and Fink (2009) for the fusiform gyrus and intraparietal sulcus ROIs. They reported larger GM in the left posterior part and less GM in the left anterior part of the fusiform gyrus of synesthetes (max *t* = 2.99 and *t* = 2.90, respectively, pSVC < 0.05). Less GM was also observed in left MT/V5 (*t* = 3.13, pSVC < 0.05). They did not correct for multiple comparisons across several ROIs. A whole brain analysis at a liberal threshold (uncorrected *p* < 0.005, corresponding to *t* > 2.68) revealed larger GM in synesthetes in three additional regions. The largest difference (*t* = 3.59) was observed in the right precentral gyrus.

Our conclusion: no statistically significant GM differences in the whole brain analysis. Weak increases and decreases of GM both inside and outside regions of interest were reported.

***Hupé 2012: 10 grapheme–color synesthetes and 25 controls, VBM-GM and WM, cluster extent statistics.*** In a VBM analysis at 3T (10 synesthetes vs. 25 controls) on the whole brain (pFWE < 0.05, images were thresholded at *p* < 0.0001; significant clusters had a minimal extent of 70 mm^3^; 6 mm FWHM smoothing; gender age and brain size were used as covariates) the authors (Hupé et al., 2012b) found no difference in GM between synesthetes and controls but higher local volume of WM in synesthetes located bilaterally in the retrosplenial gyrus (left side: pFWE = 0.019, max *t* = 5.65; right side, pFWE > 0.05, max *t* = 5.25) and in the depth of the left superior temporal sulcus (STS; pFWE = 0.075, max *t* = 6.17). There was, however, no correlation with the strength of synesthetic associations measured in each synesthete. No higher local GM or WM volume was observed in controls.

Our conclusion: the size of the WM increase was around 5%, with the largest voxelwise *t*-values measured at the time. This increase was yet just significant for cluster-extent statistics (left retrosplenial cortex, *p* = 0.04 if considering a two-tailed test; probably overfitted result due to thresholding procedure). Similar results were reported for 8 mm FWHM smoothing but there is a risk of false positives when smoothing is less than 12 mm. The lack of correlation with synesthetic strength makes it unlikely that this (underpowered) difference between groups was due to synesthesia.

***Melero 2013: eight grapheme–color synesthetes and six controls, VBM-GM, WM and DTI, uncorrected voxelwise statistics.*** Melero et al. (2013) measured structural differences at 3T using VBM (SPM8) on both T1-weighted and DTI images (15 directions). They used age, gender, and intracranial volume as covariates and computed non-parametric *t*-statistics (SnPM). They only reported uncorrected whole brain results at a very lenient threshold (*p* < 0.001; 4 mm FWHM smoothing for all analyses). They observed no difference in the fusiform gyrus and IPS at that threshold. Only at uncorrected *p* = 0.01 did they observe larger local GM volume in synesthetes in the vicinity (<8 mm) of the left caudal IPS as reported by Weiss and Fink (2009).

Our conclusion: no reported statistically significant differences between synesthetes and controls. Groups were small ([1, Sample size]), smoothing was not sufficient and non-parametric tests did not allow inferences on central tendencies.

***O’Hanlon 2013: 13 grapheme–color synesthetes and 11 controls, VBM-GM, WM and DTI, voxelwise statistics.*** O’Hanlon et al. (2013) measured structural differences at 3T between synesthetes and control women, using T1-weighted and DTI images (15 directions, *B*-value = 800 smm^-2^; *B*-value was 1000 smm^-2^ in all other DTI studies reported here). They used VBM (FSL) to compute GM and WM measures (5.75 mm FWHM smoothing; age and brain size were not used as covariates). They computed in MRIcron^12^ Brunner Munzel statistics on ranks, weighted for differences of variances (pFDR < 0.01, minimum cluster size = 10 mm^3^). For FA analysis, they used FSL to create skeleton images and performed Brunner Munzel statistics on voxels within tracts using MRIcron (pFDR < 0.01, minimum cluster size = 5 mm^3^; no covariate).

They observed larger values of GM, WM, and FA in synesthetes compared to controls, no larger values in controls. Both cortical and subcortical structural differences were observed and were largely distributed over the whole brain; no particular locus emerged across the three measures. They reported nine regions of GM increase, six of WM increase, and 14 regions of FA increase. Maximum *z*-scores were up to 5.75 (GM, left lateral occipital cortex/precuneus, 0.9 cm^3^), 7.86 (WM, right occipital pole/cuneus, 5.8 cm^3^) and 5.80 (FA, right subgyral – superior longitudinal fasciculus, 6 mm^3^).

Our conclusion: as concluded by the authors, the only consistency in those results is larger values for synesthetes. They used state-of-the-art analysis tools but the combination of low spatial smoothing and FDR statistics in small groups may have led to a high rate of false positives (see Hupé, 2015: third section “Pitfalls of MRI Statistics”).

***Zamm 2013: 10 music-color synesthetes and 10 controls, DTI, ROI statistics.*** Using DTI (30 directions) at 3T on 10 controls and 10 music–color synesthetes, Zamm et al. (2013) focused their study on two WM pathways that pass through both temporal and occipital regions, i.e., inferior frontal–occipital fasciculus (IFOF) and inferior longitudinal fasciculus (ILF). They computed a mean global FA along each pathway for each subject and for the right and left hemispheres. They observed for the IFOF a significant interaction between side and group, which could be interpreted either as higher FA in synesthetes compared to controls in the right IFOF (uncorrected *p* = 0.04) or as the presence of left/right asymmetry only in controls (uncorrected *p* < 0.01). They found no between-group FA difference in the ILF.

Our conclusion: the study tested the hypothesis of the involvement of white matter tracks traversing temporal and occipital regions. The interpretation of such a result in the IFOF is, however, difficult because the measures in the right and left hemisphere of each subject were treated as independent. A repeated-measure ANOVA would have been appropriate. The just-significant difference between small groups in the right IFOF is further undermined because differences in ROI volume or position (used as seed to identify the tracks in each subject), were not used as cofactors (the authors argued that there was no significant difference overall, but at *p* > 0.10 [2, Accepting the Null]). However, there was a significant correlation between FA in the right IFOF and mean Synesthesia Battery score of synesthetes (two-tailed *p* = 0.012; most consistent synesthetes had higher FA values), with a seven voxels cluster in the occipital part of IFOF, in the fusiform gyrus, which was more significantly correlated with the Battery score (*p* < 0.05, FWE corrected within the IFOF). We do not know how we should interpret differences of consistency in synesthetic associations (as long as the associations are constant enough to qualify as synesthetic).

***Whitaker 2014: 20 grapheme–color synesthetes and 20 controls, DTI, cluster-extent statistics.*** Whitaker et al. (2014) used DTI (six directions at 1.5 T) to compare FA between synesthetes and controls. They also analyzed all the components of the diffusion tensor model fitted at each voxel: mean diffusivity as well as the three eigenvectors; they referred to the largest eigenvalue (λ1) as parallel diffusivity and the average of the two remaining eigenvalues as perpendicular diffusivity (λ23). They performed *t*-tests and permutation-based cluster extent statistics within tracks (FSL, pFWE < 0.05, threshold-free cluster enhancement (TFCE); no spatial smoothing was indicated). They included no covariate in their analysis. Each synesthete was matched to a control of the same sex and age but they did not perform paired comparisons. They found lower FA in synesthetes compared to controls all over the brain. This decrease was associated with higher perpendicular diffusivity.

Our conclusion: cluster extent statistics were developed within the framework of the random field theory. Fiber tracks do not have the properties of a random field. Permutation tests can be applied to any kind of statistics, so why not TFCE cluster extent *t*-statistics. But no inference can be done on these results beyond the violation of exchangeability between groups (moreover, *t*-statistics are sensitive to differences of variance and outliers). The diffusion tensor model was estimated using only six directions, so even small, random variations of signal/noise ratio and artifacts in a few subjects may have caused these widespread, non-specific differences between the groups.

##### Single-case study

***Hänggi 2008: One multiple synesthete and 37 controls, DTI, VBM-GM, and WM, uncorrected cluster extent statistics.*** Hänggi et al. (2008) compared a professional female flute player, E.S., experiencing interval–taste and tone–color synesthesia, as well as perfect pitch, with a group of professional musicians (*n* = 17, 10 with perfect pitch) and a group of “normal” controls (*n* = 20). The two groups were tested on different systems and different acquisition parameters for both DTI (21 directions for musicians, 15 directions for other controls) and T1 images. E.S was scanned twice. For DTI, they used FSL for preprocessing and to create FA maps. Then they used SPM5 for smoothing and statistics. They used VBM (SPM5) for GM and WM statistics. GM and WM values were divided by the total GM and WM volume, respectively. The same statistical procedure was used in both analyses: they computed *z*-scores at each voxel by comparing E.S. values to each group variance and reported arbitrary thresholded clusters (*z* = ± 3.1, corresponding to *p* < 0.001, *k* > 50 voxels; 12 mm FWHM smoothing). They did not report whether any effect would survive a FWE correction, arguing that they had “strong *a priori* hypotheses” ([4, a hypothesis is not an *a priori*]). They also performed fiber tractography in E.S. and the 10 professional female musicians with absolute pitch perception.

They observed many structural differences (FA, WM, and GM) when comparing E.S. to controls (22/22 clusters with increased/decreased values) and to musicians (28/32 clusters with increased/decreased values), as expected given the poorly conservative statistical threshold. The critical result was the overlap of clusters across modalities and when comparing E.S. to both groups. The authors did not perform this comparison formally but they showed in their Figure 4 that there was some overlap or contiguous clusters in the region of the Heschl gyrus/planum temporale/insula, bilaterally, showing increases of both FA and WM in E.S. compared to both groups. These increases were accompanied by decreased GM in the same regions. There was therefore “a consistent pattern of bilateral brain differences in auditory areas.” The authors indicated that this was also the case in the insular cortex and occipital regions (gustatory and visual regions; no clear evidence provided). This would suggest higher connectivity between the inducing and concurrent synesthetic modalities. Tractography around the Heschl gyrus revealed indeed higher connectivity probabilities in E.S. compared to the critical control group of the 10 musicians with absolute pitch (four measures: numbers of suprathreshold probabilistic tract voxels and mean probabilistic connectivity distribution values, on the left and right sides). The largest difference was obtained for mean probabilistic connectivity on the right side, but it reached only *z* = 1.93, corresponding to *p* = 0.027 for a one-tailed test (*p* = 0.054 if making the Null hypothesis of no difference [4, a hypothesis is not an *a priori*]; *p* = 0.086 if using the appropriate Student distribution with nine d.o.f.; pFWE = 0.34).

Our conclusion: E.S. seemed to show increases of WM and FA (coherent tracts) in areas involved in the processing of the inducing stimuli (auditory area). These differences could be related to absolute pitch rather than synesthesia since the critical comparison with musicians with absolute pitch was not conclusive.

#### CONNECTIVITY STUDIES

##### Synesthetes vs. Controls

***Hänggi 2011: 24 grapheme–color synesthetes and 24 controls, structural MRI (3T), cortical thickness.*** Hänggi et al. (2011) investigated the topology of the structural brain networks in the subjects analyzed by Jäncke et al. (2009) for structural differences (see Appendix: Structural Morphometry Studies). Using cortical thickness covariations as an indicator of connectivity, they constructed connectivity matrices from region-wise cortical thickness correlations between predefined anatomical regions (brain parcellization obtained using FreeSurfer suite) and computed a set of structural networks (graphs). A surface template, onto which the surface model of each individual was realigned, was first parceled into 154 anatomical regions across each hemisphere and then refined to obtain finer parcels (1187 and 1179 in each left and right hemisphere, respectively). Cortical thickness correlation was computed for each pair of parcels leading to two connectivity matrices (154 × 154 or 2366 × 2366 size) for both synesthetes and controls. Derived networks were constructed where nodes (154 or 2366) represented anatomical parcels and edges weighted connections between parcels. To compute these weights, connectivity matrices were thresholded at different values leading to different networks (16) with different number of edges per group depending on cortical thickness correlation values. Two specific networks were also computed (132 nodes) to investigate specifically fusiform gyrus and intraparietal sulcus areas. All networks were analyzed according to the small-world theory (Watts and Strogatz, 1998). Several metrics for network characterization were computed including path length and clustering coefficient. Node centrality, measured by weighted degree and betweenness centrality, to assess the importance of a node for information integration, and hierarchical clustering, to assess network modularity, i.e., structuration in different modules composed of interconnected nodes, were analyzed. They used two-tailed *t*-tests to compare all these values between both groups (Bonferroni correction). The many measures obtained on the two network sizes were not fully consistent, rendering the global message difficult to synthesize. According to the authors, the central finding was that grapheme–color synesthetes presented a reduced small–world network organization compared to non-synesthetes, reflecting a less optimal network organization with increased clustering and hyperconnectivity. The connectivity alteration (hyperconnectivity) in grapheme–color synesthetes seemed not restricted to intraparietal sulcus and fusiform gyrus. The results even suggested hypoconnectivity within the fusiform gyrus of synesthetes (a result not compatible with the hypothesis of excessive cross wiring between color and grapheme areas in synesthetes).

Our conclusion: whatever its interpretation in terms of connectivity, the analysis of covariations of local cortical thickness throughout the brain requires that it is not biased by thickness differences between synesthetes and controls. However, such differences existed. Hänggi et al. (2011) reported that “right global cortical volume and left and right global cortical surface areas were increased in synesthetes compared with non-synesthetes.” They disregarded these differences because they did not survive Bonferroni correction. This means that these differences, probably due to random sampling, were not representative of the population but they were real and could affect all further measures ([2, Accepting the Null]). Furthermore, Jäncke et al. (2009) had reported local differences between groups in 23 regions, with higher values in synesthetes. These differences did not survive corrections for multiple comparisons, suggesting that they were also due to random sampling. As many local increases in controls (they did not test them) due to random sampling were likely. All these local differences between both groups may have biased all the statistics of connectivity to an unknown degree.

***Jäncke 2011: 12 grapheme–color synesthetes and 13 controls, EEG resting state.*** Jäncke and Langer (2011) studied resting-state of so-called colored-hearing synesthesia using 30-channels EEG (this study was part of the experiment by Beeli et al., 2008; see Appendix: EEG, MEG). Subjects rested for three successive periods with eye open (20 s) then eyes closed (40 s). Only eye-closed periods were analyzed. They first computed the averaged power spectrum in each channel, in 2 s epochs and in four frequency bands comprised between 6.5 and 21 Hz. They found no between-group differences (all uncorrected *p* > 0.1, 120 comparisons). Then, they estimated electrical sources that generated brain activity during relaxed state in the two groups with sLORETA. They considered 84 anatomical ROI (Brodmann areas based) and computed correlation measures between each ROI for each subject leading to 84 nodes network. They identified the thresholds (of the average correlation matrix that defined weighted connections – edges – between nodes) that produced the best small-world network organization across all subjects and within each frequency band. They applied these criteria to each subject and compared small-worlds indices (similarly to Hänggi et al., 2011) in both groups. There was no difference between synesthetes and controls (all uncorrected *p* > 0.2, 12 comparisons). Finally they measured in each of the 84 areas (“hubs”) the “degree” measure, which quantifies connectedness with other brain areas. They measured higher degree in synesthetes in 10 regions (uncorrected *p* < .05, 84 regions * 4 frequency bands = 336 comparisons). They did no test the reverse contrast (336 unreported comparisons). They insisted on the four differences (two-tailed uncorrected *p*-values between 0.01 and 0.04) in regions where they had expectations, in the left parietal (in theta, alpha1, and alpha2 frequency bands) and auditory cortex (AC; beta band) – among the 24 comparisons where they expected differences. They observed no difference in the fusiform gyrus. Eight other significant differences were observed in regions where they had no *a priori* (0.003 < *p* < 0.05). Their hypothesis-driven approach led them to suggest that parietal regions constitute a hub more functionally interconnected in synesthetes than in controls.

Our conclusion: Jäncke and Langer (2011) clearly acknowledged that “because of the relative small number of subjects and extremely large number of variables, it is nearly impossible to conduct classical statistical inference test” because no effect would survive the necessary correction for multiple comparisons. They therefore reported “descriptive measures of between-group differences.” These measures may be useful when confronted to other data collected using a similar methodology. No stronger inference can be made on their analysis based on *a priori* hubs localized in the parietal lobe, auditory, and visual cortex as long as there is no proof that these regions are involved in synesthesia. The differences were in any case weak and did not even survive corrections for the 24 comparisons for which they had *a priori* hypotheses. Other differences were reported involving different hubs, making this result poorly specific. The main result is, in fact, the surprisingly similar results obtained in both groups of subjects for nearly all measures, and especially the absence of connectedness difference in the fusiform gyrus.

***Dovern 2012: 12 grapheme–color synesthetes and 12 controls, fMRI (3T) resting state.*** Dovern et al. (2012) considered group independent component analysis (ICA) to identify, from 10-min long resting-state functional MRI data, intrinsic connectivity networks (ICN) potentially relevant to grapheme–color synesthesia. They identified 25 independent components. They computed a multiple spatial regression analysis to select ICNs whose associated spatial maps included the visual cortex (V1, V2, V3, V4, or V5), the auditory or the intraparietal cortex (regions identified using Anatomical Toolbox; Eickhoff et al., 2005). They identified seven ICNs involving one of these regions and present in both groups (12 controls and 12 grapheme–color synesthetes). They measured significant differences of functional connectivity between synesthetes and controls in a few voxels within the spatial maps associated with these seven networks (SPM5 ANOVA, voxelwise pFWE < 0.05). The largest differences were observed in clusters within the right and left frontoparietal networks, with both increases and decreases of functional connectivity in synesthetes compared to controls. Functional connectivity was higher in synesthetes in the few other significant voxels within the other networks.

Then, they derived inter network functional connectivity for each subject by calculating pairwise, two-tailed, correlations between the BOLD signal time courses of each ICN. Fifteen significant (pFDR < 0.05) correlations between the time courses of the seven ICNs were present in synesthetes and only five in controls. They observed that the visual networks were interconnected to the other ICNs in synesthetes but not in controls ([2, Accepting the Null]). Yet, only two network connections were significantly stronger in synesthetes than controls (two-tailed *t*-tests, pFDR ∼ = 0.05 over the 42 possible connections between the seven ICNs): the connections between both the medial and lateral visual networks and the right frontoparietal network (*p*∼ = 0.002). Critically, the connection strength of the lateral visual ICN with the auditory ICN was correlated with the individual consistency scores (rate of consistent responses over 129 items, which corresponds therefore to the number of graphemes or words with synesthetic colors); Dovern et al. (2012) reported *p* = 0.006, using a one-tailed test; we recomputed *p* = 0.011 using the appropriate two-tailed test. They also reported a positive correlation between the lateral visual ICN and the right frontoparietal ICN (two-tailed *p* = 0.07).

Additionally, a seed-based functional connectivity network was computed in each individual by computing the correlation between each brain voxel and cytoarchitectonically defined bilateral V4 regions. Right parietal cortex and bilateral auditory cortices were significantly connected to V4 in synesthetes but not controls, while regions of the frontal, temporal, and parietal cortex were significantly connected to V4 only in controls. However, the direct comparison between synesthetes and controls revealed no difference surviving correction for multiple comparisons (pFWE < 0.05).

Our conclusion: this study suggests slightly different functional connectivity in synesthetes and controls during resting state. There were, however, a few methodological issues. Respiratory and cardiac signals are potential artifacts in resting-state fMRI but were not measured. As in many resting states experiments, “subjects were instructed to keep their eyes closed but remain alert and awake during the resting-state measurements in the scanner.” In our experience, many subjects tend to fall asleep during resting state recordings even when instructed to keep their eyes open (as monitored by eye-tracking); during debriefing subjects do not systematically report falling asleep when these periods are short. On a statistical level, the increased connectivity in synesthetes reported by Dovern et al. (2012) is not grounded. First of all, they selected only seven ICNs based on which regions they supposed to be relevant to grapheme–color synesthesia. All connections within and between the 25 ICNs should have been analyzed since we showed that there was no data supporting their hypotheses. Moreover, the largest differences between synesthetes and controls were observed in the frontal–parietal networks, where there is no independent evidence so far of involvement in synesthesia. Increases and decreases of similar magnitude were observed. These differences being unlikely related to synesthesia, this calls for a hypothesis-free study of all possible connections. Dovern et al. (2012) insisted on the increased connectivity between ICNs. Only two connections were significantly stronger in synesthetes, both involving the fronto-parietal network. Significance would not survive correction for multiple comparisons if considering the 600 possible connections between the 25 and not only 7 selected ICNs. In addition, these between ICNs differences involved the fronto-parietal ICN where differences had been observed within the ICN, which may have contributed to the observed between networks differences. The critical point is whether any difference between synesthetes and controls is related to synesthesia or other random differences between these small groups of people. The critical result was, therefore, the positive correlation measured between the number of synesthetic associations and the connection strength between the auditory and the lateral–visual network. The correlation was, however, weak (*p* = 0.011, only 12 subjects) and would not survive multiple comparisons if considering all possible correlations even only within the 42 ICNs connections considered by Dovern et al. (2012) This connection was also only slightly stronger in synesthetes than controls (uncorrected *p* = 0.05).

***Neufeld 2012b: 14 tone–color synesthetes and 14 controls, fMRI (1.5T) during auditory stimulation.*** Neufeld et al. (2012b) performed a functional connectivity analysis on the fMRI data obtained by Neufeld et al. (2012a); see Appendix: Functional Studies; Group Studies, Auditory Stimuli). They computed connectivity using only three seeds: the left IPC and the bilateral AC. In the IPC they had observed stronger BOLD signal in synesthetes than controls for tones vs. baseline while the AC responded more to tones in both controls and synesthetes. Their IPC region was 3 cm away from the parietal ROI put forward by Jäncke and Langer (2011; see **Table 2**). They averaged the beta series across voxels within a 5-mm sphere around the center of mass of these clusters and computed the correlation with the beta series of all other brain voxels. They performed cluster-extent statistics (SPM5, voxel threshold p < 0.001; minimal extent was 41 voxels, corresponding to pFWE < 0.05 as estimated “based on permutation analyses of sub-samples of a large dataset”; 8 mm FWHM smoothing). They detected no cluster of increased connectivity in controls. They detected significant clusters of increased connectivity in synesthetes between the right AC and the left and right motor cortex, as well as between the left IPC and both the left primary AC and the right primary visual cortex. There was no increased connectivity between the visual and AC. They considered that these results supported the disinhibited feedback model of synesthesia.

Our conclusion: we agree with the authors that these results are more compatible with the disinhibited feedback theory than the cross-activation theory. However, we are not sure that the false positive rate was controlled enough with the permutation method (see Hupé, 2015: third section “Pitfalls of MRI Statistics,” paragraph on Permutation tests) especially when using only unspecified sub-samples of the whole data set. The main issue is the strong dependence of the results on the definition of the ROIs. In this review we reveal that there is no evidence yet of the involvement of the parietal cortex in synesthesia. Moreover, there is no consistency across all studies about the exact location of this area. The authors observed a stronger correlation in synesthetes between parietal cortex and V1, not V4 or color sensitive areas of the fusiform gyrus. This observation is probably compatible with synesthetes paying more attention to stimuli. Neufeld et al. (2012b) proposed no explanation for the stronger correlation observed between the auditory and motor cortex. This unexpected, unexplained, result makes the other correlations less specific, and suggests that many more differences may have been found between synesthetes and controls if investigating other seed areas.

***Sinke 2012: 18 grapheme–color synesthetes and 18 controls, fMRI (1.5T) during visual stimulation.*** Sinke et al. (2012) performed an analysis similar to Neufeld et al. (2012b) on grapheme–color synesthetes. The functional results of this study were described in the “Functional Studies” section of the Appendix: “Group Studies, Visual Stimuli.” The seeds for the connectivity analysis were defined the same way as in Neufeld et al. (2012b). For the contrast between synesthetes and controls, they found stronger BOLD signal in the left IPL. This region was, however, 16 mm away from the parietal seed identified by Neufeld et al. (2012b; **Table 2**). When contrasting letters and pseudo-letters across all subjects they found three significant clusters in the right hemisphere, one in the visual cortex and two in the parietal cortex. Statistics were similar to Neufeld et al. (2012b; cluster-extent statistics using SPM5, voxel threshold *p* < 0.0001; minimal extent was 51 voxels, corresponding to permutation computed pFWE < 0.05). They computed functional connectivity for stimulation with letters and pseudo-letters separately (this was an event-related design; they used the beta weights). They found stronger connectivity in synesthetes between the left IPL and the right and left visual cortex for letters but not pseudo-letters. However they did not contrast the two conditions ([2, Accepting the Null]) and if they had they may have found no difference since maximal *t*-values were similar (max *t* = 4.64 for letters and max *t* = 4.22 for pseudo-letters). They found no significant connectivity differences between groups and for both stimuli when using the seeds identified by the main factor ‘letters.’ When considering both stimuli together they obtained a similar cluster to the one revealed when using the left IPL (for letters), this time when the seed was in the right IPL. The part of the visual cortex revealed in both cases was in BA18, right on the surface of the occipital pole. There was no stronger connection in synesthetes involving V4.

Our conclusion: this study brings further evidence against the cross-activation theory as well as the possible involvement of color areas in grapheme–color synesthesia. The conclusions are, however, limited because of the dependence on the definition of the ROIs. The evidence in favor of the disinhibited feedback theory is very weak: even more than in the study by Neufeld et al. (2012b) the parietal region was not specific of the synesthetic experience since activation was observed for both inducing and non-inducing letters. The connectivity difference was also probably not specific to the synesthetic experience. Finally, the localization of the “significant” voxels right along the surface of the occipital cortex let us suspect that they could be artifactual.

***Tomson 2013: 20 grapheme–color synesthetes and 19 controls, fMRI (3T) during rest and audiovisual stimulation.*** Tomson et al. (2013) explored functional connectivity with fMRI in synesthetes and controls during four successive conditions: 3 min of eye-closed rest, 5.3 min eye-closed while listening to the audio track of children’s television clips (Sesame Street), 7.1 min freely watching Sesame Street (audiovisual condition), and again 3 min of eye-closed rest. Sesame Street segments were rich in graphemes in order to elicit synesthesia.

They analyzed functional connectivity between nine ROIs supposed to be involved in processing graphemes and colors. Using the data of a first fMRI experiment (see Appendix: Functional Studies; Group Studies, Visual Stimuli) on 16 synesthetes (five of them participated in the second experiment) and 15 controls, they defined as a “grapheme ROI” a bilateral region of the lateral posterior inferior temporal, curiously identified as containing voxels that responded more to pseudographemes than graphemes in all three group contrasts (controls, synesthetes, and collapsed). Based on coordinates from the literature they created 9 mm diameter spheres in the left Visual Word Form Area (Cohen et al., 2002) and in bilateral V4, VO1, and VO2 (Brewer et al., 2005), considered as color ROIs. They also performed a hypothesis-free analysis using the anatomical AAL atlas to parcel the entire brain in 90 regions (Tzourio-Mazoyer et al., 2002), to which they added their nine ROIs (there was therefore some anatomical overlap).

They quantified the correlations between ROI time series during the four conditions. They compared the network sparsity (optimal number of edges) of each group (permutation test). They found no sparsity difference when testing the 99 regions (*p* = 0.86). When restricting the analysis to the nine grapheme and color ROIs, they observed more edges in synesthetes than controls^13^ in the first (but an equal number in the second) rest condition (28 vs. 22 edges) as well as in the audio (not the audiovisual) condition (21 vs. 15 edges). They observed that in the audio condition synesthetes had significantly more edges (7 vs. 2) connecting grapheme and color nodes than controls, leading them to suggest that synesthetic connectivity shifted to “greater density of connections in Rest to a greater density of connections between color and grapheme regions in Audio,” thus favoring synesthetic associations. Such interpretation is, however, not consistent with synesthetes having 10 edges between grapheme and color nodes in rest and only seven in Audio, as well as with the absence of group differences in the audiovisual and the second rest conditions.

They also computed graph theory metrics over the 99 regions and found no difference between groups for the indices of modularity, degree, betweenness centrality, and local efficiency. They explored in detail modularity: the number of modules (interconnected nodes) was not different between groups, but the identity of the modules could be different. In each group (time series concatenated across subjects) they identified by bootstrapping (resampling with replacement) the correlations between nodes that were invariant to the ordering of time points. When comparing co-occurrence frequencies (computed for 100 within time-series resamples) across nodes over the whole brain (only including co-occurrence >50%, over the 99*98 possible couples), only slightly larger values were observed in controls (0.05 < pFDR < 0.1 for six co-occurrence in the audio and one in the audiovisual condition, no difference in the rest conditions). When restricting the analysis to co-occurrence frequencies calculated at 15 nodes (their nine regions of interest and six regions extracted from the synesthesia literature: bilateral SPL, bilateral lingual gyri, and bilateral middle frontal gyri) they observed larger values (pFDR < 0.1) this time in both controls and synesthetes, again in the audio and audiovisual, not rest, conditions. The pattern of differences suggested that synesthetes cluster visual regions more tightly than controls (0.015 < pFDR < 0.1 for all but one connection), but without specific differences between color and grapheme regions.

Our conclusion: this experiment had similarities with the resting state fMRI study by Dovern et al. (2012) and also used tools for network analysis similar to Hänggi et al. (2011). When computing global network metrics without any *a priori* they did not find any difference between groups. When restricting the analysis to regions supposed to be involved in grapheme–color synesthesia, Tomson et al. (2013) reported a few differences between groups. The results for the analysis of connectivity between predefined “color” and “grapheme” nodes were not consistent. Moreover, the nodes were not defined in a consistent way. For example, there exists no localizer for a “grapheme area,” and no evidence in the literature that such localized region exists, especially when collapsing letters and numbers. Here, anyway, the reverse contrast was used (voxels responding less to graphemes that shapes labeled as pseudographemes, containing more edges – see Appendix: Functional Studies; Group Studies, Visual Stimuli). Also, there is no evidence that retinotopic areas V4, VO1, and VO2 can be reliably identified based on anatomical coordinates. The modularity analysis suggested that synesthetes cluster visual regions more tightly than controls in the presence of stimulation potentially triggering synesthetic associations. The differences were, however, just significant even though FDR correction was applied only “based on the number of hypotheses tested per NOI” (Node of Interest); if they had used FWE correction adjusted for the 15 NOIs (therefore pFWE < 0.0033) these differences would not be significant anymore. Respiratory and cardiac signals were not measured. Interestingly, no evidence was found in favor of a role of parietal regions or suggesting connectivity differences between color and grapheme regions, even for this uncorrected ROI based analysis.

***Volberg 2013: seven grapheme–color synesthetes and seven controls. EEG during visual stimulation.*** Volberg et al. (2013) compared the intertrial phase coherence of EEG signals during stimulation with inducing vs. non-inducing graphemes in seven synesthetes and controls ([1, Sample size]). Phase coherence reflects the local synchrony of neural signals, a measure related to local functional connectivity. They also measured phase-locking values between distant electrodes (64 electrodes system), a measure possibly related to distant neuronal synchrony (as long as the signals from the electrodes are independent, which cannot be completely the case for EEG signals).

For local synchrony, they computed at each frequency if the *number of electrodes* with significant differences at any time point (uncorrected *p* < 0.05) was larger than expected by chance (two-tailed pFWE < 0.05, permutation of labels). In synesthetes, they measured larger phase coherence for inducing graphemes within the lower beta band (nine electrodes when six was the maximum value for 97.5% of the permutations) at 410 ms. This phase coherence observed at occipital and frontal electrodes was also significantly larger than measured in the control group. Source reconstruction suggested the involvement of the visual cortex, including the left fusiform gyrus. Phase coherence was larger for non-inducing stimuli in the gamma range at 360 ms (eight electrodes when six was the maximum value for 97.5% of the permutations); the difference with controls, observed at central electrodes, was also significant. These late effects suggest that they do not have a causal role in the experience of synesthesia.

Volberg et al. (2013) also measured an increase of long-range couplings in the alpha range, mostly between the left parietal and occipital electrodes, which occurred earlier (100–120 ms) for inducing than non-inducing stimuli (16 couplings when 9 was the maximum value for 97.5% of the permutations; two-tailed pFWE < 0.001; the number of significant pairings among 1953 was computed at uncorrected *p* < 0.005). There was also a later (280–540 ms) decrease in the number of couplings in the theta frequency band. Both effects were significant when compared to controls.

Volberg et al. (2013) suggested that the decrease of long-range coupling within the theta range was compatible with a decrease of inhibition for inducing letters in synesthetes, leading to an increase of local synchrony in the beta band in the visual cortex.

Our conclusion: the authors observed a rather complex pattern of significant differences in their second-level statistics, while using systematically as first-level statistics non-reliable paired *t*-test because based on only seven values ([1, Sample size]). If using non-parametric test, significance could not go below 0.02; yet they used uncorrected *p* < 0.005 for long-range couplings, meaning that their results rely strongly on the hypothesis of Normality, a hypothesis impossible to verify (most first level significant statistics may be driven by only 1 or 2 subjects). Other choices in the analysis strategy were problematic, and the choice of paradigm was questionable: within a Posner task, they included the presentation of only one grapheme inducing a synesthetic color or one non-inducing symbol. These 1.4^∘^ stimuli were presented for only 150 ms, at 7.5^∘^ eccentricity, with no guaranty of the induction of a synesthetic color (no phenomenological report was requested). They argued observing an interference in the response times of the main task, demonstrating that induction did happen. However, the critical comparison with controls was far from significant (*p* = 0.18). The results were based on the analysis of the seven synesthetes only, based on permutations (significant effects only reflect that exchangeability of labels is violated). The comparisons with the control group were performed only for results significant in synesthetes (more differences between controls and synesthetes could have been observed, making the results much harder to interpret).

##### Individual differences among synesthetes

***van Leeuwen 2011: 15 grapheme–color synesthetes, fMRI (3T), DCM.*** van Leeuwen et al. (2011) used dynamic causal modeling (DCM) to test whether the different subjective experiences of 10 projectors (six of them refined as “mental screen projectors”) and five associators ([1, Sample size]) are due to differences in effective connectivity within the synesthesia network. These synesthetes were included in their previous fMRI study (van Leeuwen et al., 2010). Two DCM models were considered following the cross-wiring (direct connections between grapheme and color areas) or the disinhibited feedback (aberrant feedback from parietal lobe to color area) theories. The three nodes of the corresponding networks, right fusiform gyrus (considered as “V4, color area”), left SPL and “left fusiform gyrus” (LSA, letter shape area) were selected from fMRI data. LSA was obtained with the contrast [false fonts > non-inducing graphemes], collapsed across all subjects (*N* = 38, 19 synesthetes and 19 controls). Bilateral activations were measured in the fusiform and occipital gyri. Only one seed of the left side was used. Its coordinates correspond to the area called “Left inferior occipital gyrus” by van Leeuwen et al. (2010), even though it was called here “left fusiform gyrus”. A left “V4 color area” was identified when contrasting synesthetes and controls for the contrast [inducing graphemes > non-inducing graphemes; pSVC = 0.052, see our report of the functional results of van Leeuwen et al., 2010], even though van Leeuwen et al. (2010) had argued against the coactivation of V4 by real and synesthetic colors. The relevant “color contrast” [colored graphemes > black graphemes] in the 38 subjects resulted in bilateral activation in the fusiform gyri but it was not used ([6, selective reporting]). The left SPL was identified when contrasting synesthetes and controls for the contrast [inducing (black) graphemes > colored graphemes]. They tested the different models in 15 synesthetes only (they could not extract time series due to lack of activation in four synesthetes and they did not report results for controls [6, selective reporting]). They found that across the 15 synesthetes there was no strong preference for either the bottom–up or the top–down model. The bottom–up model was, however, better for projectors and the top–down model better for associators. Mental screen projectors (also called strong associators) had intermediate preferences between both models.

Our conclusion: though very suggestive, these results should be interpreted with caution because of the small sample size and the arbitrary and questionable choice of the nodes for the DCM analysis. The so-called LSA was obtained for the contrast false-fonts minus graphemes, because the opposite, designed, contrast did not reveal any significant voxel. The “color” seed was not obtained using the color localizer. The left SPL region, crucial for the analysis, was not obtained with the contrast specific to synesthesia (inducing graphemes > non-inducing graphemes). The used contrast (black inducing graphemes > colored non-inducing graphemes) is difficult to interpret when comparing synesthetes and controls. The MNI coordinates were different from other reported parietal regions (**Table 2**; there is anyway no strong evidence in favor of the involvement of the parietal cortex: see the Discussion of main text, Part 1, “Parietal cortex?”). Group comparisons between synesthetes were not done “everything else being equal” because each subject had different stimuli (tailored to each synesthete). Groups of controls matched to synesthetes could and should have been tested against the DCM models. Intrinsically, the DCM approach can only confirm *a priori* predefined models while other options not initially included cannot be tested. As long as the tested models are largely questionable (see main text, Part 1) the interpretation of these results is also questionable.
